# Differential Role of the T6SS in *Acinetobacter baumannii* Virulence

**DOI:** 10.1371/journal.pone.0138265

**Published:** 2015-09-24

**Authors:** Guillermo D. Repizo, Stéphanie Gagné, Marie-Laure Foucault-Grunenwald, Vitor Borges, Xavier Charpentier, Adriana S. Limansky, João Paulo Gomes, Alejandro M. Viale, Suzana P. Salcedo

**Affiliations:** 1 Bases Moléculaires et Structurales des Systèmes Infectieux, CNRS UMR 5086, Université Lyon 1, Institut de Biologie et Chimie des Protéines, Lyon, France; 2 Unité de Microbiologie, Adaptation et Pathogénie, CNRS UMR 5240, Université Lyon 1, Villeurbanne, France; 3 Bioinformatics Unit, Department of Infectious Diseases, National Institute of Health, Lisbon, Portugal; 4 Instituto de Biologia Molecular y Celular de Rosario (IBR, CONICET), Departamento de Microbiologia, Facultad de Ciencias Bioquimicas y Farmaceuticas, Universidad Nacional de Rosario, Rosario, Argentina; Academia Sinica, TAIWAN

## Abstract

Gram-negative bacteria, such as *Acinetobacter baumannii*, are an increasing burden in hospitals worldwide with an alarming spread of multi-drug resistant (MDR) strains. Herein, we compared a type strain (ATCC17978), a non-clinical isolate (DSM30011) and MDR strains of *A*. *baumannii* implicated in hospital outbreaks (Ab242, Ab244 and Ab825), revealing distinct patterns of type VI secretion system (T6SS) functionality. The T6SS genomic locus is present and was actively transcribed in all of the above strains. However, only the *A*. *baumannii* DSM30011 strain was capable of killing *Escherichia coli* in a T6SS-dependent manner, unlike the clinical isolates, which failed to display an active T6SS *in vitro*. In addition, DSM30011 was able to outcompete ATCC17978 as well as *Pseudomonas aeruginosa* and *Klebsiella pneumoniae*, bacterial pathogens relevant in mixed nosocomial infections. Finally, we found that the T6SS of DSM30011 is required for host colonization of the model organism *Galleria mellonella* suggesting that this system could play an important role in *A*. *baumannii* virulence in a strain-specific manner.

## Introduction

The *Acinetobacter* genus comprises a heterogeneous group of strictly aerobic Gram-negative bacterial organisms endowed with large metabolic versatility and including pathogenic and non-pathogenic environmental species. *Acinetobacter baumannii* is frequently associated with disease in the context of hospital-acquired infections, implicated in severe ventilator-associated pneumonia, bacteraemia and surgical site or wound infections [1]. Infections due to multi-drug resistant (MDR) *A*. *baumannii* strains, in particular carbapenem-resistant, are an increasing burden on health care facilities. *A*. *baumannii* shows a remarkable ability to acquire genetic elements [2] and is inherently resistant to disinfection and desiccation [3]. Biofilm formation enables its persistence on abiotic surfaces such as medical equipment, a true impediment to its eradication in the clinical setting [4]. Numerous factors have been implicated in formation of biofilm in *A*. *baumannii* and adherence to abiotic surfaces, namely the CsuA/BABCDE chaperone-usher pili assembly system [5]. The O-glycosylation system has also been implicated in early stages of biofilm formation as well as virulence in model organisms commonly used to assess virulence of *A*. *baumannii* strains, such as the larvae from the insect *Galleria mellonella* and a mouse model of peritoneal sepsis [6,7]. Additional factors have been associated with biofilm formation such as the outer membrane protein A [8], which also plays a role in adherence to host cells during infection [8]. The ability of *A*. *baumannii* to adhere to biotic surfaces such as lung epithelial cells also constitutes an essential aspect of its virulence [9].

Recently, several groups have shown the presence of functional T6SS in different *Acinetobacter* species [10–12]. The T6SS, known to inject protein effectors across both prokaryotic and eukaryotic target cell membranes [13], provides a colonization advantage in the environment or during infection for many pathogens. More recently, T6SS-mediated killing has also been shown to lead to DNA release and enhanced horizontal gene transfer events which may contribute to spread of antibiotic resistance [14]. The T6SS consists of a contractile bacteriophage sheath-like structure, an assembly of different proteins forming a needle or spike structure used to penetrate the target cell. In the T4 bacteriophage this structure encompasses the gene products gp5 and gp27, which associate into a huge complex in which the distinctive needle-like shape is provided by the C-terminal domain of gp5 [13]. In bacterial T6SSs the equivalent proteins are fused into a large protein known as VgrG, in which the gp27 and gp5 folds are represented by the N-terminal and C-terminal domains, respectively [13]. The dynamic activity of the T6SS leads to the secretion of proteins that comprise the spike/tube complex, VgrG and Hcp, with the concomitant disruption of the envelope of the target cell with potentially lethal effects. Some VgrG proteins, designated as evolved VgrGs, also display a number of C-terminal domains that may act as effectors or bind specific effector proteins adding more diversity to the T6SS roles in cell-cell interactions [12,13,15].

The role of T6SS in *Acinetobacter* is still unclear: while the T6SS of the pathogenic species *A*. *nosocomialis* M2 and the environmental strain *A*. *baylyi* were shown to mediate killing of *Escherichia coli* laboratory strains [11,12] in the case of *A*. *baumannii* ATCC17978 this system was neither implicated in bacterial competition nor virulence [10]. Recent comparative analysis of the genomes of phylogenetically- and epidemiologically-related MDR *A*. *baumannii* clinical strains indicated the complete loss of the T6SS genomic locus in isolates of a particular clade [16,17]. This additionally suggests that this system is not absolutely necessary in the conditions prevailing in the clinical setting.

In this study we carried out a comparative analysis of *A*. *baumannii* strains ATCC17978 and DSM30011 as well as MDR clonally-related strains implicated in outbreaks in hospitals in Argentina [18]. We found significant differences between these strains regarding T6SS functionality. Interestingly, although apparently endowed with all necessary gene components in their genomes, it does not seem that the MDR clinical strains tested have a fully active T6SS. In contrast to the ATCC17978 and clinical strains, the T6SS of DSM30011 mediated killing of *E*. *coli*, other *A*. *baumannii* and several pathogens often associated with nosocomial infections and was found critical for virulence *in vivo* in the *Galleria mellonella* model organism.

## Materials and Methods

### Bacterial cultures

Bacteria were routinely cultured in liquid Tryptic Soy Broth (TSB) or Tryptic Soy Agar (TSA). Antibiotics, when appropriate, were added to *Acinetobacter* culture media at the following concentrations: apramycin 30 μg/ml, gentamicin 50 μg/ml, kanamycin 50 μg/ml, and tetracycline 10 μg/ml. For *E*. *coli* cultures antibiotics were added at the following concentrations: ampicillin 100 μg/ml, apramycin 50 μg/ml, nalidixic acid 20 μg/ml, and kanamycin 20 μg/ml. Bacterial strains used are summarized in S1 Table.

### Biofilm assays

Adherence assays were performed in 24-well polystyrene microtitre plates, as previously described [19]. Bacteria were grown in BM2 minimal medium [20] supplemented with 10 mM potassium glutamate (BM2G) as the carbon source. Cultures performed in quadruplicates were inoculated at an initial Abs_600_ of 0.1 from an overnight culture grown in TSB, and subsequently incubated under static conditions for 24 h at 37°C. One of the wells for each strain was thoroughly resuspended and used to measure Abs_600_. Attached bacteria to the plastic surface on the three remaining wells were stained with 1% Crystal Violet for a period of 15 min and rinsed twice with water. The dye attached to the bacteria was solubilized in 400 μl 70% ethanol, to which 600 μl of water were added and Abs_580_ was measured to quantify the levels of Crystal Violet. The Abs_580_/Abs_600_ ratio was used to normalize the amount of biofilm formed to the total cell content of each sample tested, to account for biofilm variations due to differences in bacterial growth. All assays were done in triplicates using fresh samples each time.

For biofilm imaging, overnight cultures of *A*. *baumannii* strains were re-inoculated into glass-bottom 24-well plates containing BM2G medium. The plates were incubated at 37°C for 24 h under static conditions and then labelled with anti-*A*. *baumannii* antiserum or DAPI. Images were taken with a confocal Zeiss 710 microscope (z-stacks) and analysed with ImageJ.

### RNA isolation and RT-PCR

For total RNA isolation, *A*. *baumannii* strains were grown overnight in 2 mL of TSB medium and then sub-cultured (1/100 dilution) up to Abs_600_~1. The cells were then harvested by centrifugation at 4°C and total RNA was extracted with the RNeasy Mini kit. Subsequently, the QuantiTect Reverse Transcription kit was used to remove DNA from the samples, and synthetize the corresponding cDNAs from 1 μg of total RNA. HcpF/HcpR and TssMF/TssMR primers sets (S2 Table) were used to detect transcripts corresponding to *hcp* and *tssM* genes, respectively. The RNA not subjected to RT was also run in PCR (negative control) to ensure that PCR positive reactions were due to the presence of transcripts and not to contaminating DNA. The *rpoB* transcripts were used as endogenous control (S2 Table).

### Construction of mutant and complemented strains

All plasmids and primers used in this study are listed in S1 and S2 Tables. Two DNA fragments encompassing each 500 bp flanking the *A*. *baumannii* ATCC17978 *tssM* gene were PCR-amplified using primers 17UpF and 17UpR (upstream region) and 17DoF and 17DoR (downstream region), which were designed after the sequences of *tssM* and neighboring regions of this strain available on databases (A1S_1302 and A1S_1303). These segments were cloned into a SmaI pre-digested pEX100T/Kan plasmid using the In-Fusion commercial kit (Clontech) and following manufacturer indications. The resulting plasmid, pEX-TssM17 was electroporated into *A*. *baumannii* ATCC17978 and unmarked *tssM* deletion mutants were obtained following the protocol described by Hoang & Schweizer [21]. Deletion of the *tssM* gene was verified by PCR using primers 17ChUp and 17ChDo.

For the construction of a *tssM* (DSM30011_11660) deletion mutant of *A*. *baumannii* DSM30011, fragments of 2 kb flanking this gene were amplified using the primers DSMUpF and DSMUpR for the upstream region, and DSMDoF and DSMDoR for the downstream region. Subsequently, the amplimers were digested with EcoRV (restriction sites are carried in the sequence of DSMUpR and DSMDoF), blunt-ligated through this site and cloned into pGEM^®^-T Easy using A-overhangs remaining at the other side of each amplimer, originating pGEM-2.1. In parallel, an apramycin resistance cassette was amplified from *E*. *coli* MFDpir chromosomal DNA (accession number X01385) using primers ApraF and ApraR. The PCR product was digested with EcoRV and ligated to pGEM-2.1 plasmid, producing pGEM-2.2, which carries the flanking regions of DSM30011 *tssM* gene interrupted by an apramycin resistance cassette. The whole construct was next excised from pGEM-2.2 by restriction with SmaI and directly electroporated into DSM30011 competent cells followed by selection on apramycin-containing TSA plates. Proper replacement of the *tssM* gene with the apramycin resistance cassette was confirmed by PCR using primers DSMChUp and DSMChDo. For the construction of a *tssM*-expressing plasmid for complementation purposes, the *tssM* gene from *A*. *baumannii* DSM30011 was amplified using primers CTssMF2 and CTssMR2. The PCR product was cloned into the BamHI site of pX5K, so that the *tssM* is placed under control of the *Ptac* promoter. The pTssM resulting plasmid was introduced by electroporation into the DSM30011 Δ*tssM* strain for complementation studies. All constructions were verified by sequencing at the GATC-Biotech service.

### Hcp Secretion analysis

*A*. *baumannii* strains were inoculated into 50 ml of TSB or L-broth at an Abs_600_ of 0.08 and incubated at 37°C until Abs_600_~1.0 or prepared as described for the competition assays (see below). Supernatants were obtained by centrifugation of cultures at 4,000 x *g* followed by filtration (0.22 μm) and a 50-fold concentration using an Amicon Ultracel 3K centrifuge filters. Concentrated cell supernatants and whole cell lysates were resuspended in 40 mM Tris pH 8, 200 mM NaCl, 5% glycerol and analyzed by 18% SDS-PAGE. The identity of excised 19 KDa bands assigned to Hcp from the SDS-PAGE analysis of supernatants or total cell extracts was done by LC-MSMS at the Protein Science Facility, SFR Biosciences Lyon. When needed, the resolved proteins were detected with an anti-Hcp antibody against *Pseudomonas aeruginosa* Hcp [22] at a dilution of 1/ 500, provided by A. Filloux (Imperial College London, UK) or anti-EF-Tu at 1/1000, given by R. Voulhoux (CNRS UMR7255 and Aix-Marseille University, Marseille, France).

### Bacterial competition assays

Experiments were done essentially as described previously [11]. Briefly, the different *A*. *baumannii* and *E*. *coli* strains were grown overnight in 2 mL of L-broth (10 g/l tryptone, 5 g/l yeast extract and 0.5 g/l NaCl) medium and then sub-cultured in the same medium (1/100 dilution) up to Abs_600_~1. Cultures were then diluted in L-broth to Abs_600_ of 0.4 and the different bacterial strains were mixed in 1:1 or 10:1 ratios (predator:prey), as indicated. Aliquots of 20 μl of these mixtures were laid on the surface of L-medium supplemented with 1.5% Agar and incubated at 37°C for 4 h. The bacterial spot on the agar surface was subsequently removed, vigorously resuspended in PBS, serially diluted and plated onto solid selective media. For *E*. *coli* DH5α, selective medium contained 20 μg/ml nalidixic acid or 50 μg/ml apramycin for strains transformed with pGEM-2.2. For *Acinetobacter* strains, the selective media employed are indicated in the figure legends. A mini-Tn7 [23] was used to incorporate a cassette conferring gentamicin resistance into the chromosome of the DSM30011 strain whereas the mini-CTX1 harbouring a tetracycline resistance cassette [21] was used to confer tetracycline resistance to the ATCC17978 strain. For selection of *P*. *aeruginosa* strains PAK (a gift from A. Filloux, Imperial College London), PA14 and PA7 (given by Romé Voulhoux, CNRS UMR7255 and Aix-Marseille University, France) selective *Pseudomonas* PIA media was used. For *Klebsiella pneumoniae* strain 52145 (kindly provided by J. Bengoechea, Queen’s University, Belfast, UK) media with rifampicin at 25 μg/ml was used. All strains grew to similar population densities regardless of co-incubation with *E*. *coli*; starting inocula where always verified by CFU counts (data not shown). In order to test the dependence of killing on contact, assays were also performed by interposing a 0.22 μm pore-membrane in between *A*. *baumannii* strain 17978 laying on the solid medium and *E*. *coli* DH5α on the top of the filter. After incubation, the membrane was lifted from the plate and treated as described above.

For all bacterial competition experiments one representative image is shown and CFU counts included for a minimum of 3 independent assays to enable accurate assessment of experimental variation.

### *Galleria mellonella* infection assays

*G*. *mellonella* larvae were purchased from Sud Est Appats (http://www.sudestappats.fr/) and were used the day after arrival. Groups of twenty randomly picked larvae were used for each assay condition. The different *A*. *baumannii* strains tested were grown overnight in TSB and then diluted with PBS to obtain the CFU/ml titers (as indicated in the corresponding figure legends), which were verified by colony counts on TSA for all inocula. A Hamilton microliter syringe was used to inject 10 μl of the bacterial suspensions into the hemolymph of each larva via the second last left proleg. As a control, one group of *G*. *mellonella* was injected with 10 μl of PBS. After injection, the larvae were incubated in plastic plates at 37°C and the numbers of dead individuals were scored regularly. Survival curves were plotted using PRISM software, and comparisons in survival were calculated using the log-rank Mantel-Cox test and Gehan-Breslow-Wilcoxon test.

### Competitive index

The DSM30011 strain and its Δ*tssM* mutant were separately grown overnight in 5 ml of TSB medium at 37°C with shaking. Bacterial cultures of parental (apramycin-sensitive) and mutant (apramycin-resistant) strains were subsequently diluted in PBS to 10^8^ CFU/ml, mixed in equal volumes and 10^7^ CFU in 100 μl aliquots were inoculated intraperitoneally into 6 week-old C57BL/6 female mice. Input ratios (apramycin-resistant versus apramycin-sensitive bacteria) were determined by plating ten-fold sequential dilutions on TSA. After 18 h post-inoculation spleens were aseptically removed, weighed and homogenized in 1 ml of PBS. Several dilutions were plated on TSA with or without apramycin to determine the output ratios. The competitive index (CI) corresponds to the ratio between the output of Δ*tssM* mutant and the wild-type DSM30011 strain divided by the ratio between the input of the mutant and the wild-type strain. Animals were purchased from Charles River and housed in the PBES animal facility. The care and use of experimental animals complied with local animal welfare laws and guidelines. Euthanasia was carried out by cervical dislocation when necessary, if established early endpoints were reached (loss of mobility and more than 20% weight loss) during the experiment with animals monitored every 4 h. The protocols were approved by the CECCAPP ethical committee *(* *C* *omité d’* *E* *valuation* *C* *ommun au* *C* *entre Léon Bérard*, *à l’* *A* *nimalerie transit de l’ENS*, *au* *P* *BES et au laboratoire* *P* *4)*.

### Accession numbers

The GenBank accession numbers of the *tss* locus and the four putative *vgrG* genes of the Ab244 clinical strain are KR109191, KR155241, KR155242, KR155243 and KR155244, respectively. The corresponding DNA sequences of the Ab242 and Ab825 strains are identical to those of Ab244. The GenBank accession numbers of the *tss* locus and the four putative *vgrG* genes of the DSM30011 strain are KT334324, KT334325, KT334326, KT334327, and KT334328, respectively (see S3 and S4 Tables for details). The DNA sequence of the DSM30011 genome is currently under deposit in GenBank. RAST annotation server and NCBI databases were used for homologues searches and conserved domain detection. Pfam homology searches were done at http://pfam.xfam.org/search

### Statistical analysis

All statistical analysis was performed using GraphPad Prism version 6.0 for Mac, GraphPad Software, La Jolla, California, USA. The *Galleria* survival statistical analysis was done using the log-rank Mantel-Cox test and the Gehan-Breslow-Wilcoxon test; for biofilm assays one-way ANOVA was carried out to compare all means. CI was analysed using a homoscedastic and 2-tailed Student's t-test, with the null hypothesis: mean index is not significantly different from 1.0. P<0.05 were considered significant and coded as follows: **** for *P*<0.0001, *** for 0.0001<*P*<0.001, ** for 0.001<*P*<0.01 and * for 0.01<*P*< 0.05.

## Results

### Identification of genes encoding a T6SS and associated components in the strains under study

In view of the importance of T6SS in bacterial competition and as a virulence factor in a number of opportunistic pathogens, we investigated its role in a panel of *A*. *baumannii* strains currently being studied in our laboratory: ATCC17978, an extensively used *A*. *baumannii* strain which was initially isolated from the cerebrospinal fluid of a hospitalized patient more than 60 years ago [24]; DSM30011, a poorly characterized environmental strain which was shown to be naturally competent and to efficiently kill *G*. *mellonella* larvae [25]; and Ab242, 244 and 825, three clonally-related MDR *A*. *baumannii* clinical strains implicated in outbreaks in hospitals of Argentina [18]. The main difference described for these three clinical strains is the presence of different alleles of the outer membrane protein CarO (including a disrupted allele for Ab825), which mediates the influx of carbapenem β-lactams and basic amino acids through the outer membrane [18,20,26].

**Fig 1:**
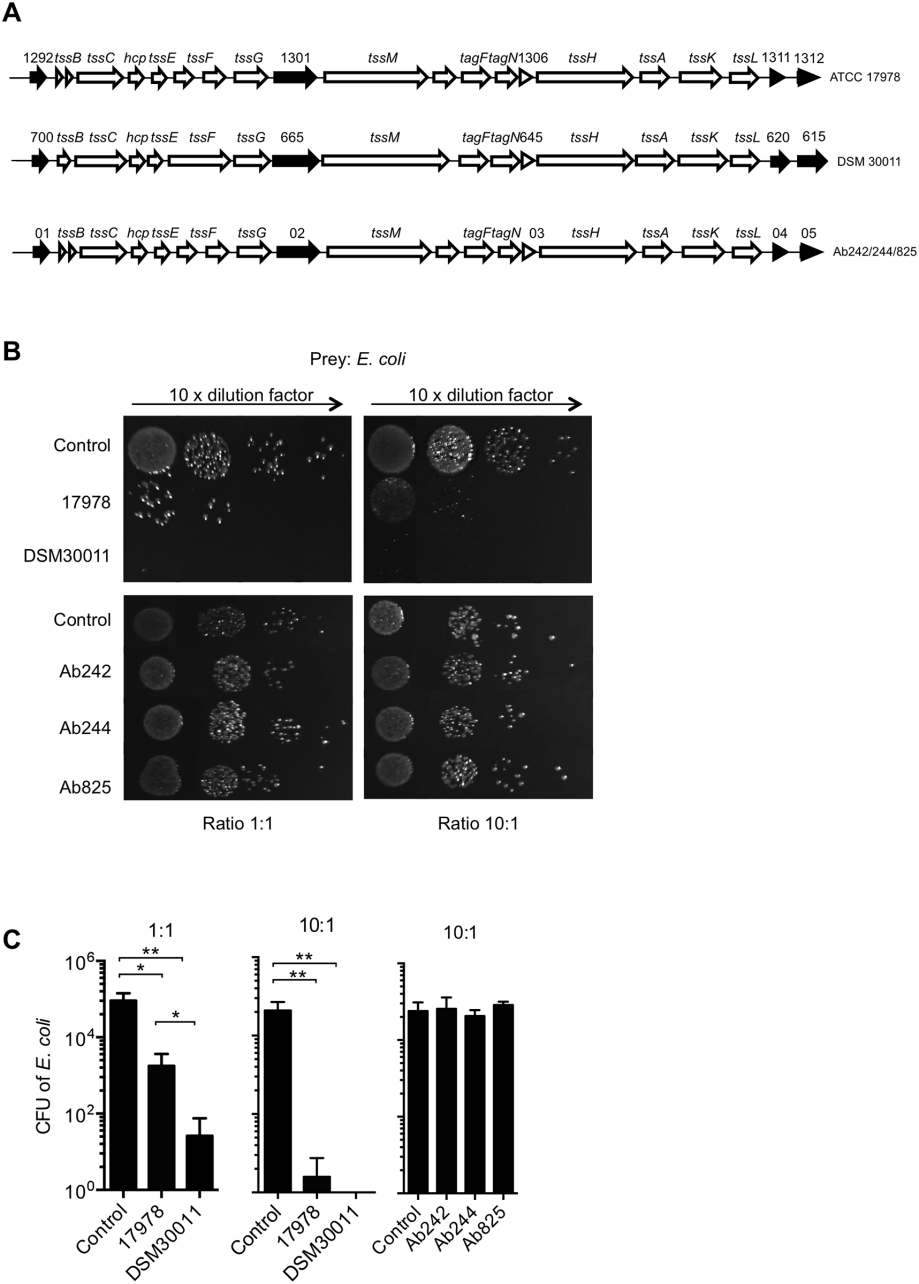
Ability of different *A*. *baumannii* strains to out-compete *E*. *coli*. A) Comparative analysis between the T6SS gene loci of *A*. *baumannii* ATCC17978, DSM30011 and clinical strains. Black arrows indicate genes specific to *Acinetobacter*. B) Survival of DH5α *E*. *coli* after incubation in growth medium (control) or with *A*. *baumannii* ATCC17978 or DSM30011 at the indicated *A*. *baumannii*: *E*. *coli* ratios (top panel). Bottom panel corresponds to survival of apramycin-resistant DH5α *E*. *coli* after incubation in growth medium (control) or with Ab242, Ab244 or Ab825 strains at the indicated ratios. Images correspond to one representative experiment from a minimum of three independent assays quantified in C) to visualize experimental variation, expressed as means ± SDM plotted in a logarithmic scale.

The genomes of the DSM30011 and the MDR strains were sequenced and analysed for T6SS genes. This search revealed the presence in the genome of each of these strains a single cluster of 18 T6SS genes similar to that previously described in other *Acinetobacter* species [10,11], from which four are specific to the genus (Fig 1A, black arrows, see also S3 Table). The DNA sequence of the *tss* loci of the Ab242/244/825 clinical strains were identical between them. Sequence comparison analyses indicated that the homologous polypeptides encoded by 17 out of the 18 genes of the *tss* clusters of DSM30011, ATCC17978, and the *A*. *baumannii* clinical strains shared an overall identity of >97% between them. The least homologous polypeptides encoded by DSM_11700/A1S_1292/Ab244_01 (Fig 1A) shared an identity of ~81% and all contained a predicted signal peptide at their N-terminal regions, but no homology could be revealed to protein domains described in databases (data not shown).

In addition to the *tss* clusters, 4 independent loci containing genes encoding putative VgrG proteins generally secreted via T6SS were found in each of the analysed *A*. *baumannii* strains (S1 Fig and S4 Table). All of these polypeptides are generally composed by N-terminal (N-t) and C-terminal (C-t) domains (S1A Fig) characteristic of VgrG proteins [12,13,15], followed in all cases by a conserved domain of unknown function (DUF) of ~150 amino acids length of the DUF2345/PF10106 family and, depending on each VrgG, of C-terminal stretches of variable amino acid composition and length. Two exceptions are formed by Ab_788 and 17978_933 VgrGs, in which these C-terminal additions are absent and part of the DUF2345 domains also contain deletions of various extents (S1B Fig). Notably, while most of the analyzed *vgrG* loci encoded full-length VgrG proteins endowed with all the above-described domains in a single polypeptide, in both ATCC17978 and the *A*. *baumannii* clinical strains one *vgrG* locus was split into two genes separately encoding N-t and C-t domains (S1B Fig). Regarding the variable extensions attached to the C-terminal domains of *A*. *baumannii* VgrGs (S1A Fig) no sequence homology could be detected at these regions (S1B Fig). This suggests that these may have additionally evolved differential functions in the different *A*. *baumannii* strains under study [12,13,15], adding further levels of complexity to the *A*. *baumannii* T6SS repertoire.

### Analysis of *A*. *baumannii* ability to compete against *E*. *coli*

Given that previous work has shown clear differences in the ability of different *Acinetobacter* species to out-compete *E*. *coli* [10–12], we investigated this phenotype for our *A*. *baumannii* strains. The different *A*. *baumannii* strains were mixed with *E*. *coli* at 1:1 and 10:1 ratios and after 4 h of incubation, bacterial dilutions were spread in nalidixic acid plates which select for *E*. *coli*. We observed that both *A*. *baumannii* ATCC17978 and DSM30011 strains are able to kill *E*. *coli* (Fig 1B and 1C). This assay was repeated using the clinical strains at 1:1 and 10:1 ratios. However, since the MDR strains are resistant to nalidixic acid we used an *E*. *coli* derivative strain carrying a plasmid with an apramycin resistance cassette. Surprisingly, we observed that none of the *A*. *baumannii* MDR strains were capable of out-competing *E*. *coli* (Fig 1B, bottom panel and C).

### T6SS locus expression and differential Hcp secretion between clinical and non-clinical strains

**Fig 2:**
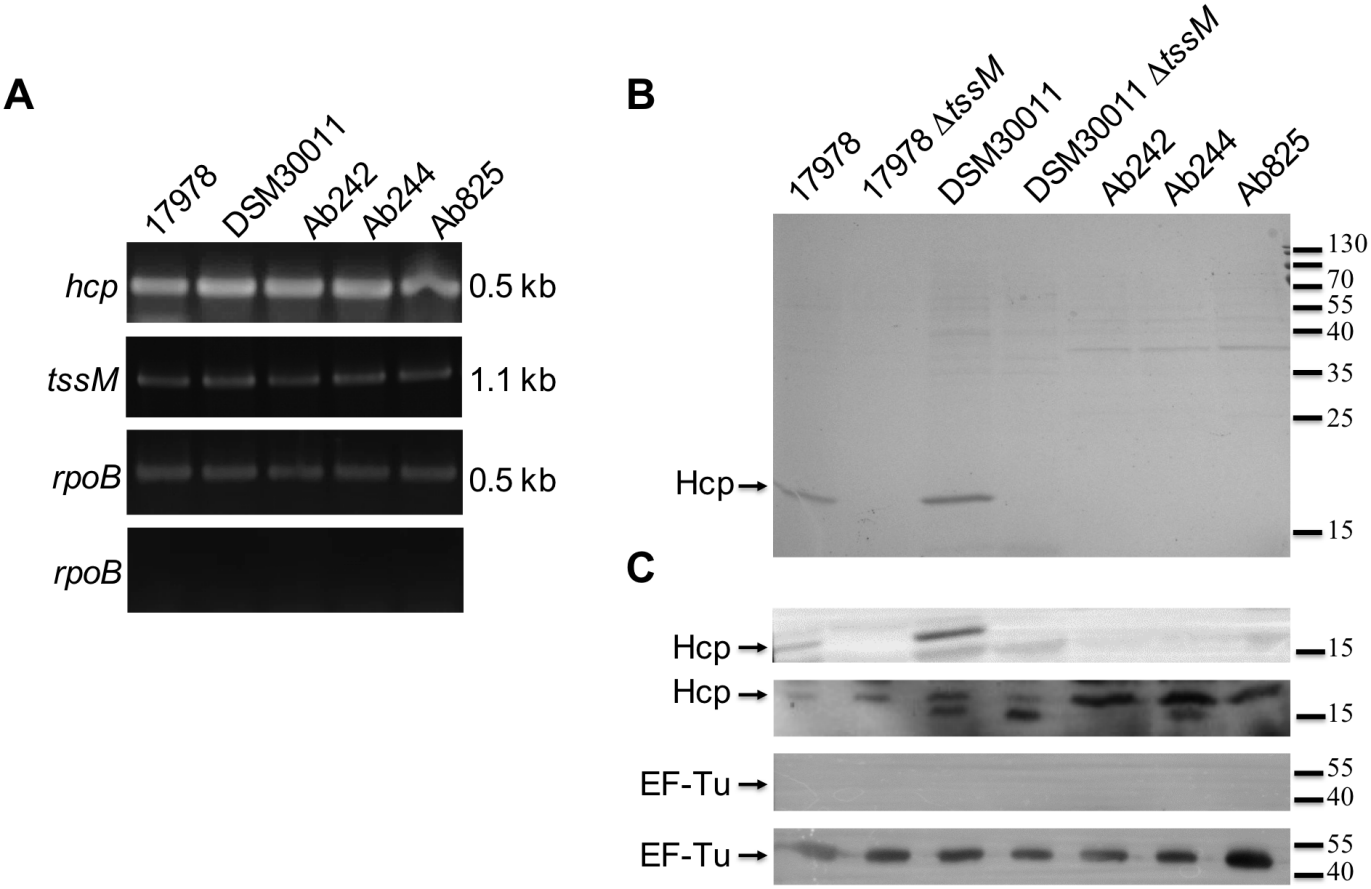
T6SS locus expression. A) RT-PCR transcriptional analysis of *hcp* and *tssM* expression in strains grown in L-broth; *rpoB* gene expression was used as endogenous control. The RNA not subjected to RT was also run in PCR (bottom panel, negative control) to ensure that PCR positive reactions were due to the presence of transcripts and not contaminating genomic DNA. B) The presence of Hcp (arrows) in concentrated culture supernatants of the indicated *A*. *baumannii* strains grown up to exponential phase in L-Broth was determined by 18% SDS-PAGE and Coomasie Blue staining. C) Control immunoblottings showing the presence of Hcp in the supernatant (first panel) and whole lysates fractions (second panel). A cell-lysis control using antibodies directed against EF-Tu (standard cytoplasmic marker) was performed using supernatant (third panel) or whole lysate (fourth panel) fractions. The final positions of the molecular mass markers (in kDa) are indicated on the right margin.

As the different *A*. *baumannii* strains tested displayed very distinct abilities to outcompete *E*. *coli* we investigated if the T6SS was functional in these strains. RT-PCR analysis confirmed that *hcp* and *tssM*, two key T6SS genes, are being actively transcribed under growth conditions used for bacterial competition assays (L-broth) in all our strains (Fig 2A). In addition, immunoblots performed with an anti-Hcp antibody [22], showed that this protein is present in whole cell lysates for all the strains (Fig 2C, second panel). The identity of the Hcp bands (19 kDa) excised from Coomassie stained gels was further verified by LC-MS analysis.

As Hcp secretion reflects the functionality of the T6SS, we then analysed supernatants obtained from exponential phase cultures of each strain. A clear Hcp band was detected for ATCC17978 and DSM30011 strains but absent in the corresponding T6SS mutants lacking TssM (Fig 2B and 2C, first panel), known to be critical for T6SS activity [10,13]. These results indicate that the T6SS is functional in the ATCC17978 and DSM30011 strains. Surprisingly, this is not the case for the clinical isolates Ab242, Ab244, and Ab825 which, in fact, can synthesize Hcp but are seemingly unable to secrete it, neither in conditions used during the killing assays (Fig 2B and 2C, first panel) nor in standard TSB culture conditions (S2 Fig). It is worth noting that sequence analysis indicated no deletions or mutations in genes coding for T6SS components in these clinical strains as compared to DSM30011 or ATCC17978 (S3 Table) that could explain the observed differences in secretion abilities between them.

### *A*. *baumannii* DSM30011 T6SS-dependent bacterial competition

**Fig 3:**
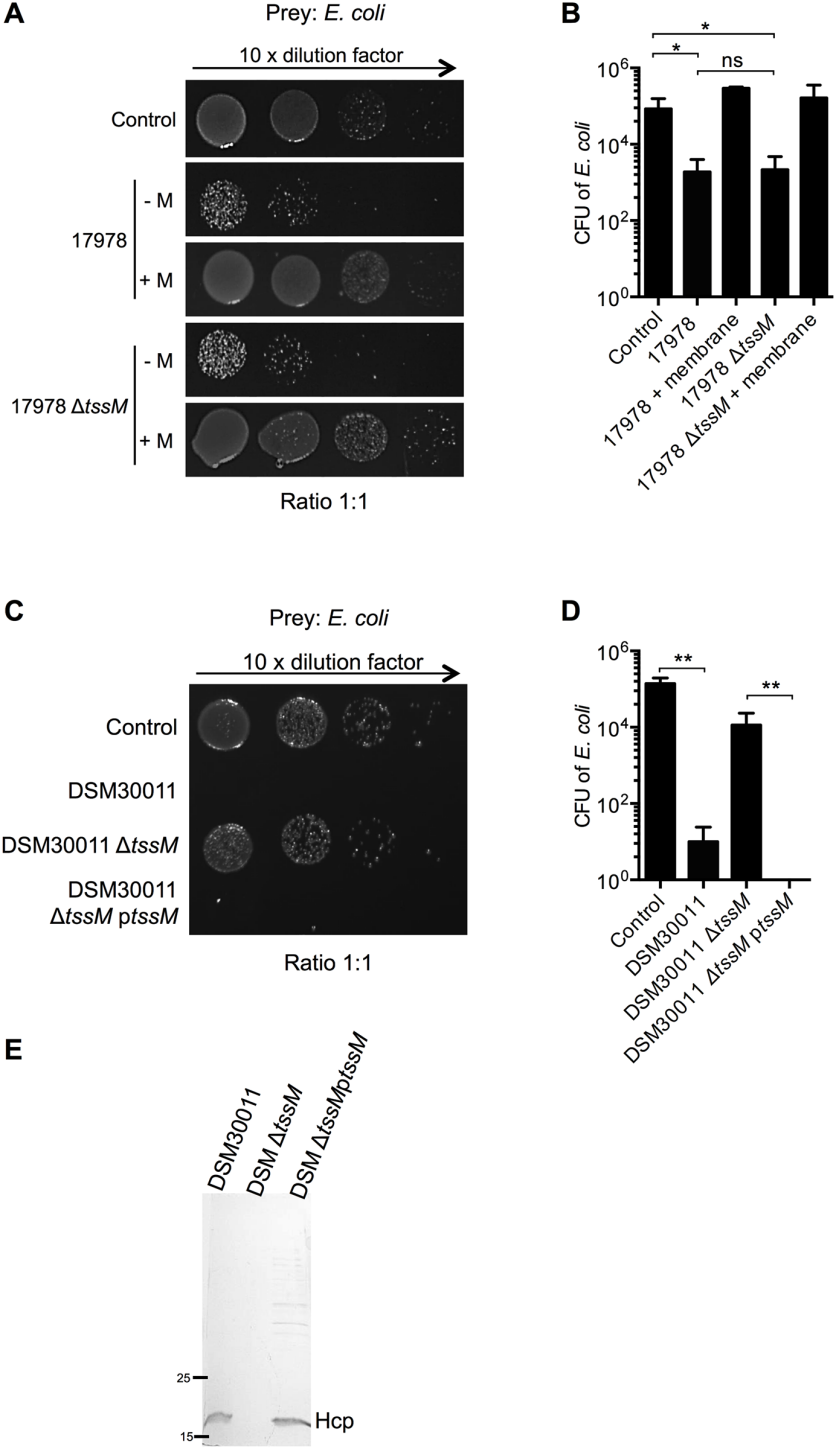
The *A*. *baumannii* DSM30011 T6SS is required for out-competing *E*. *coli*. A) Representative image showing survival of DH5α *E*. *coli* after incubation in growth medium (control) or with *A*. *baumannii* ATCC17978 wild type and Δ*tssM* strains at a 1:1 ratio with or without the presence of a membrane (+M) and B) corresponding quantification (*N* = 6). C) Survival of DH5α *E*. *coli* after incubation in growth medium (control) or with *A*. *baumannii* DSM30011 wild type, Δ*tssM* and Δ*tssM* complemented strains at a 1:1 ratio, with D) corresponding quantification (*N* = 4). E) 18% SDS-PAGE (stained with Coomassie Blue) analysis of Hcp secretion in concentrated culture supernatants of *A*. *baumannii* DSM30011 wild type, Δ*tssM* and *tssM* complemented strains grown up to exponential phase in TSB. Molecular markers are indicated on the left. All quantifications are expressed as means ± SDM plotted in a logarithmic scale.

In order to determine if the T6SS was responsible for the observed killing, Δ*tssM* deletion mutants in the ATCC17978 and DSM30011 backgrounds were tested. We observed no difference in *E*. *coli* killing for the ATCC17978 Δ*tssM* mutant with respect to the wild type (Fig 3A and 3B) consistent with what has been previously reported [10]. Interestingly, the T6SS-independent killing of *E*. *coli* by the *A*. *baumannii* ATCC17978 strain was contact-dependent as introduction of a membrane between the two strains completely abolished this phenotype (Fig 3A and 3B). It is worth noting that neither contact-dependent growth inhibition (CDI) systems nor additional T6SS were found encoded on the genome of ATCC17978.

In sharp contrast to ATCC17978, the introduction of the Δ*tssM* mutation into the DSM30011 strain completely abolished its ability to outcompete *E*. *coli* (Fig 3C and 3D) in correlation with the concomitant loss of Hcp secretion (Fig 3E). The wild-type killing phenotype and the Hcp secretion were fully restored by expressing *tssM* from a plasmid in the DSM30011 mutant strain (Fig 3C, 3D and 3E). In conclusion, *A*. *baumannii* DSM30011 killing of *E*. *coli* is dependent on a functional T6SS.

**Fig 4:**
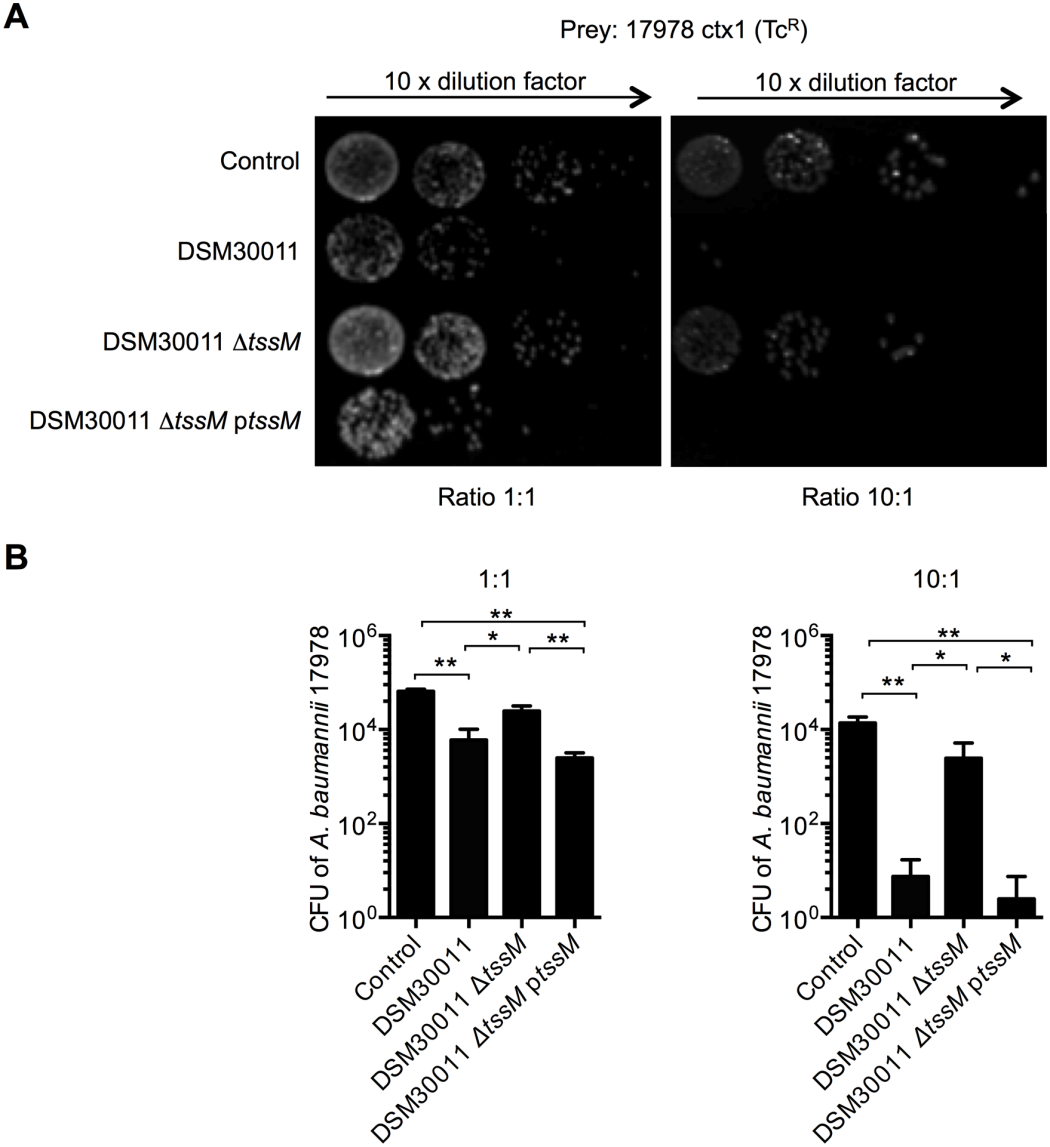
Competition between *A*. *baumannii* species. A) Survival of tetracycline-resistant (Tc^R^) ATCC17978-CTX1 strain after incubation in growth medium (control) or with the DSM30011 wild type, *tssM* deleted or complemented strains at 1:1 (left panel) or 10:1 (right panel) ratios and B) corresponding quantification (*N* = 4).

It has recently been shown that different *V*. *cholerae* strains can out-compete each other in a T6SS-dependent manner [27]. When bacteria from DSM30011 and ATCC17978 strains were co-incubated (1:1 ratio), we observed a reduction in CFU counts for the tetracyclin-resistant prey ATCC17978. This tendency increased when the strains were mixed in a 10:1 ratio (Fig 4A and 4B). This decrease was not observed with the DSM30011 Δ*tssM* deficient strain but restored in the complemented *tssM* strain (Fig 4A and 4B), showing that DSM30011 is able to use its T6SS to kill ATCC17978. When the attacker and prey were switched and mixed in a 10:1 ratio, a decrease was observed for the gentamicin-resistant DSM30011 prey when mixed with the wild type or Δ*tssM* ATCC17978 strain (S3 Fig), indicating that ATCC17978 is able to outcompete the DSM30011 strain by a T6SS-independent mechanism, as observed for *E*. *coli* (Fig 3A and 3B).

### T6SS involvement in *A*. *baumannii* competition with other Gram negative nosocomial pathogens

**Fig 5:**
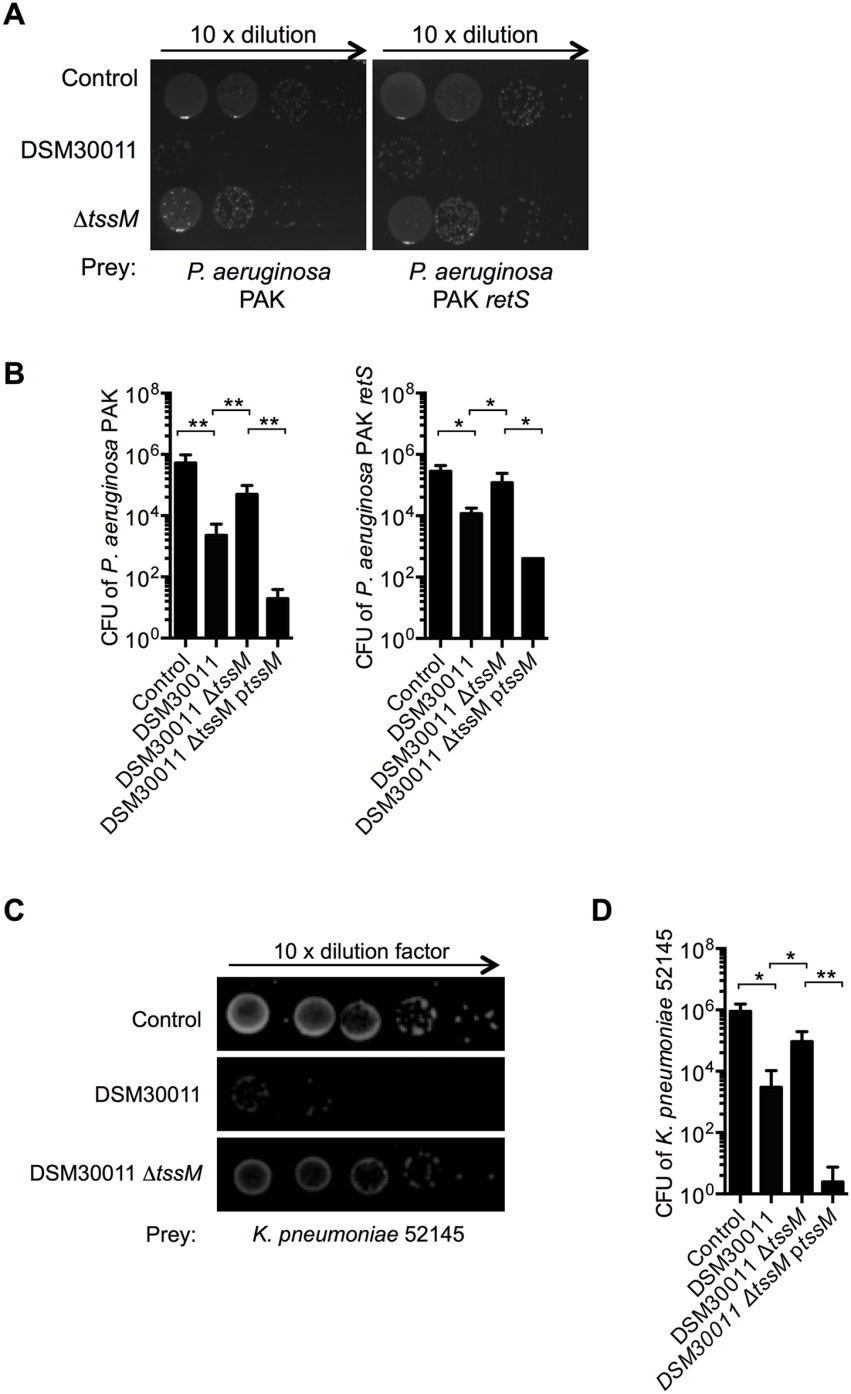
Competition between *A*. *baumannii* DSM30011 and nosocomial pathogens. Survival of A) different *P*. *aeruginosa* strains and C) rifampicin-resistant *K*. *pseumoniae* after incubation in growth medium (control) or with *A*. *baumannii* DSM30011 wild type, *tssM* deleted or complemented strains at a 10:1 ratio. *Pseudomonas* strains were selected with *Pseudomonas* Isolation Agar (PIA) media. Quantification is shown in B) and D), in which data are expressed as means ± SDM plotted in a logarithmic scale to visualize experimental variation from 3 independent experiments.

As hospital-acquired infections are often the result of colonization of patients with a mixed population of bacteria we analysed the ability of *A*. *baumannii* to kill *Klebsiella pneumoniae* and *Pseudomonas aeruginosa*, two other nosocomial pathogens associated with respiratory infections. For these experiments, we focused on the DSM30011 that showed the strongest ability to outcompete *E*. *coli*. We found that in our killing conditions, DSM30011 efficiently outcompeted the *P*. *aeruginosa* PAK strain in a T6SS-dependent manner (Fig 5A and 5B), when present at a ratio of 10:1 against its prey. This was also observed for other *P*. *aeruginosa* strains, namely PA14 and PA7 (data not shown). More interestingly, the DSM30011 could also outcompete a PAK *retS* mutant in which the T6SS is active [12] and data not shown, [28]. In addition, killing of *K*. *pneumoniae* was also observed (Fig 5C and 5D). In all cases, we consistently observed enhanced killing for the complemented strain Δ*tssM*p*tssM* confirming T6SS dependency (Fig 5B and 5D). Together these data show that the *A*. *baumannii* DSM30011 strain has the ability to kill other nosocomial pathogens.

### The T6SS of *A*. *baumannii* DSM30011 is not implicated in biofilm formation

**Fig 6:**
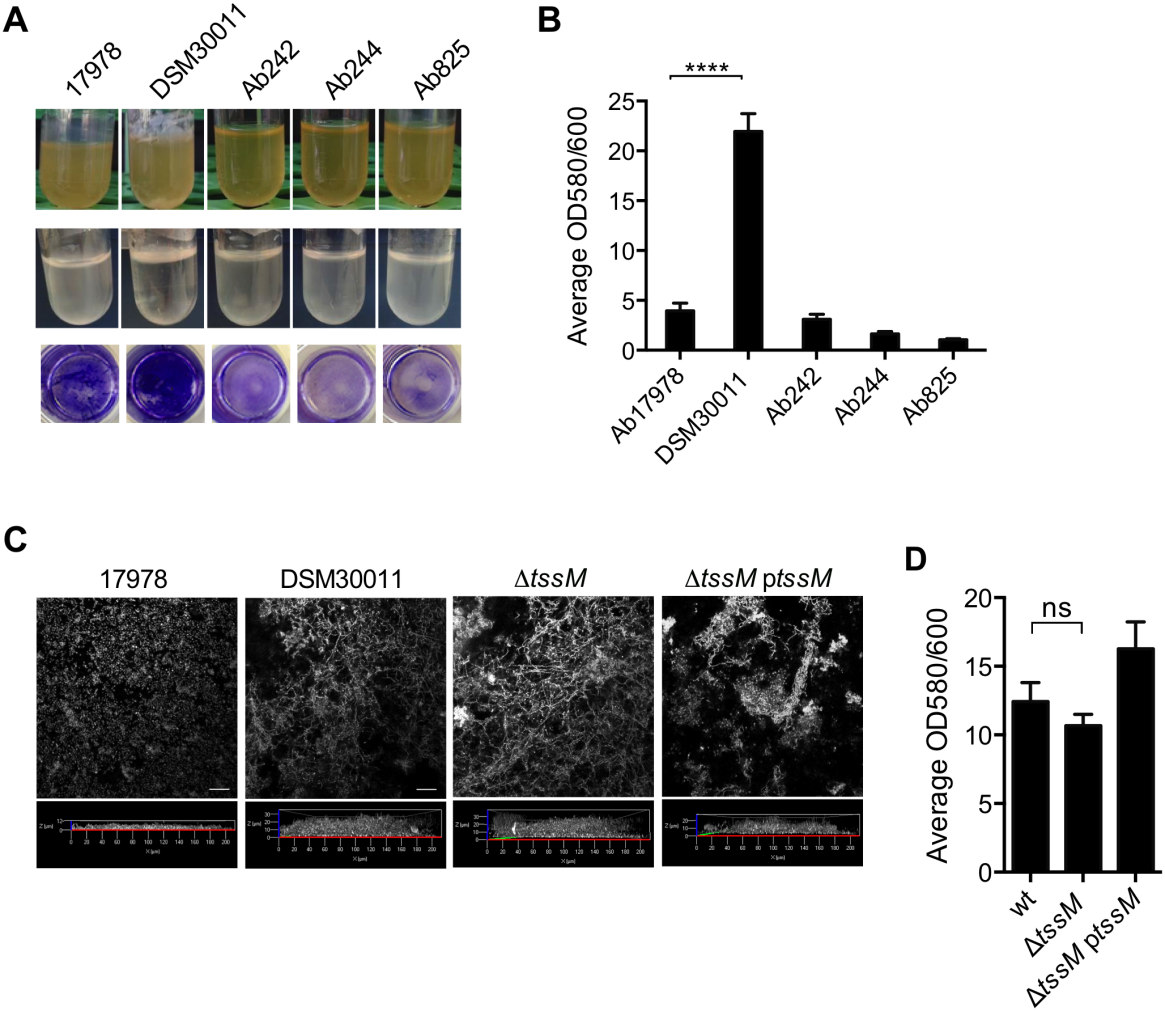
*A*. *baumannii* strain-specific growth characteristics and biofilm formation. A) The different strains were grown over-night in TSB rich medium (upper panel) or BM2 minimal medium supplemented with 10 mM glutamate (BM2G; middle panel). Bottom panel corresponds to polystyrene wells microtiter plates labelled with crystal violet and quantification is shown in B). The results are expressed as means ± SEM of experiments performed in triplicate (**** *P*<0.0001). The difference between the DSM30011 and each of the clinical isolates was also significant (**** *P*<0.0001) but not shown in the graph for simplicity. C) Confocal microscope 3D reconstruction of biofilm formed by the different strains, formed on glass-bottom slides after 24 h in static conditions. Images correspond to a slice obtained from each biofilm stack, labelled with DAPI. Depth analysis is shown for the 3D reconstruction of the z-stacks obtained for each strain. D) Analysis of the biofilm forming capacities of wild-type DSM30011, Δ*tssM*, and Δ*tssM*p*tssM*-complemented strains grown for 24 h in BM2G medium following crystal violet staining.

When routinely cultivating the analyzed *A*. *baumannii* strains in liquid culture media we repeatedly observed that DSM30011 cells have a higher tendency to grow on the liquid-air interface forming a thick pellicle on the surface. This observation was confirmed by analyzing their growth characteristics in both rich (TSB) and glutamate supplemented minimal (BM2G) liquid media (Fig 6A). We then investigated the ability of the different *A*. *baumannii* strains to produce biofilm, thought to provide a reservoir for transmission of *A*. *baumannii* to susceptible patients in hospital surfaces [4]. Following 24 h incubation, the DSM30011 strain exhibited the highest ability to adhere to polystyrene surfaces suggesting biofilm production (Fig 6A and 6B). In agreement with previous work [29], we observed that ATCC17978 is not a strong biofilm producer, a situation also observed for the clinical strains analysed which produced similar or even lower amounts of biofilm than this reference strain (Fig 6A and 6B). Confocal microscopy confirmed the ability of *A*. *baumannii* DSM30011 cells to form a biofilm with numerous large bacterial aggregates visible after 24 h (S4 Fig). On the contrary, this was not found for the ATCC17978 strain (S4 Fig) or the clinical isolates (data not shown). Analysis of the depth of bacterial growth (excluding the bacterial aggregates) on the glass-bottom plates under static growth conditions confirmed extensive biofilm formation in the case of DSM30011 in contrast to ATCC17978 cells (Fig 6C). As the T6SS has been implicated in biofilm formation in other bacterial pathogens [30–32] we investigated the ability of the *A*. *baumannii* DSM30011 Δ*tssM* mutant to adhere to abiotic surfaces and form biofilm. Under static growth conditions no significant differences were observed in comparison to the wild-type or complemented strains by neither microscopy analysis of biofilm depth (Fig 6C) nor following crystal violet staining (Fig 6D), suggesting that the T6SS is not required for biofilm formation in this *A*. *baumannii* strain.

### The T6SS of *A*. *baumannii* DSM30011 is required for virulence *in vivo*

**Fig 7:**
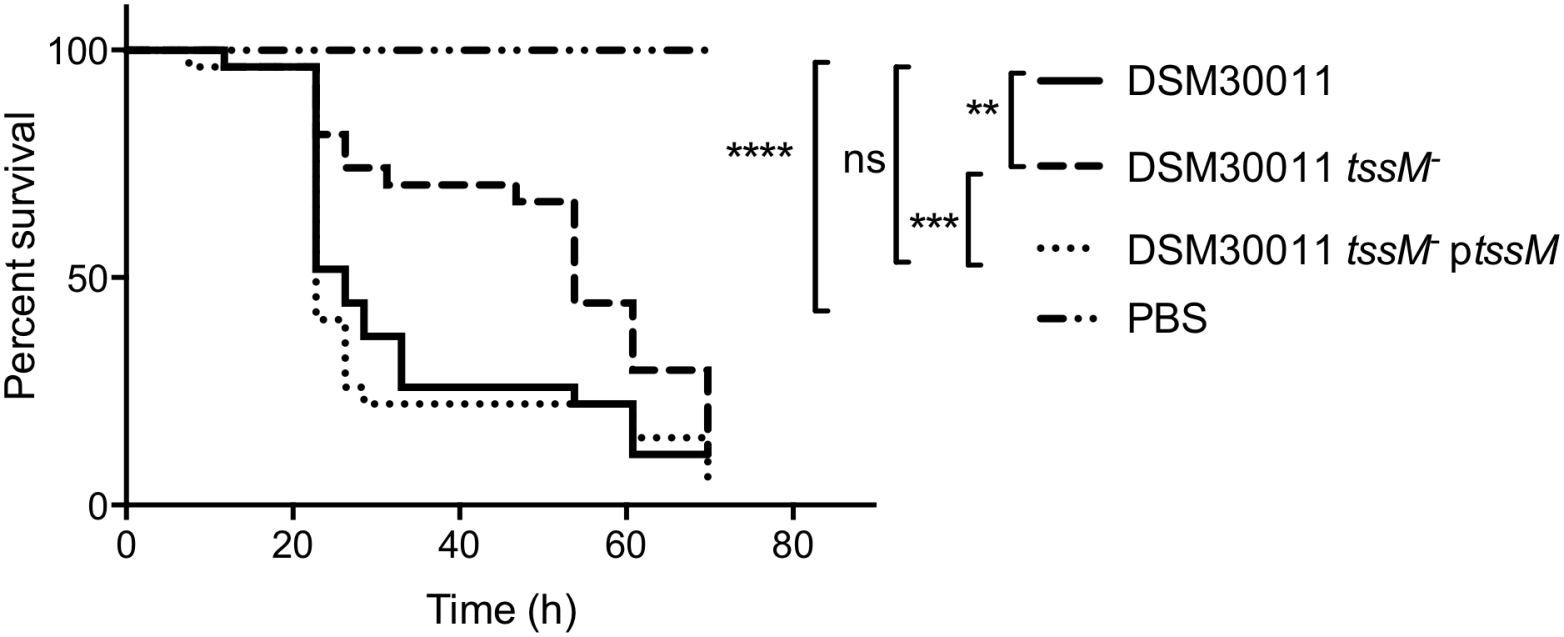
*A*. *baumanni* DSM30011 T6SS role in virulence. Comparative survival analysis of *G*. *mellonella* larvae inoculated with 5x10^5^ CFU of *A*. *baumannii* DSM30011 wild type, its isogenic Δ*tssM* mutant or complemented strain. Data are representative of three separate survival experiments, each performed with 20 larvae per strain. Survival curves were constructed by the Kaplan-Meier method and compared by log-rank analysis (* *P*<0.05; n.s.: non-significant).

In order to determine if the DSM30011 T6SS fulfils other roles beyond bacterial killing, we further characterized the corresponding Δ*tssM* strain. The involvement of the T6SS of *A*. *baumannii* DSM30011 in virulence was tested *in vivo* basing on previous established protocols [33]. We first inoculated mice with 10^8^ CFU of the wild-type DSM30011 strain and their weight and health were monitored for 2 days. We found that intraperitoneal (i.p.) inocula of 10^8^ CFU results in death of all mice in less than 24 h most likely from septic shock rather than *A*. *baumannii* colonization. On the contrary, a 10^7^ CFU injection dose was found to be sub-lethal, resulting in most mice sick and showing significant weight-loss at 18 h post-inoculation, followed by gradual recovery in the hours after. We therefore selected this dose to i.p. inject mice with a mixed inocula containing the DSM30011 Δ*tssM* and the parental wild-type strain and obtained a CI of 0.328 ± 0.0547 at 18 h post-inoculation (statistically different from 1, P = 0.008, *N = 5*). However, due to loss of the plasmid in this model of infection we were not able to complement the mutant phenotype and therefore we could not determine with certainty the role of the *A*. *baumannii* DSM30011 T6SS in mice. Therefore, no additional mouse models of infection were tested for ethical reasons and instead, to further evaluate possible roles of the T6SS in *A*. *baumannii* DSM30011 virulence, we used *G*. *mellonella* killing assays as an alternative infection model. *G*. *mellonella* has been widely used for studying the virulence mechanisms of several human pathogens, including *A*. *baumannii* [7,34–38]. In addition, the DSM30011 strain was previously shown to be virulent in this model organism [25]. We observed a significant attenuation of the DSM30011 Δ*tssM* strain that was fully reverted by the expression of *tssM* from a plasmid (Fig 7). Overall, these results show that the T6SS of the *A*. *baumannii* DSM30011 strain is implicated in virulence in contrast to the ATCC17978 strain [10].

## Discussion

This work highlights that the *A*. *baumannii* T6SS can be implicated in host colonization but in a strain-specific manner. We observed striking differences between the *A*. *baumannii* strains tested in bacterial competition assays. MDR *A*. *baumannii* strains were unable to induce killing of *E*. *coli*, due to an inactive T6SS in our laboratory conditions as demonstrated by lack of export of Hcp. Heterogeneity in Hcp export in *Acinetobacter* has been previously reported by Weber *et al*. [10], and might reflect different mechanisms of regulation of T6SS assembly. It remains possible that additional signals are necessary to induce secretion of Hcp, such as contact with a particular host target or an environmental signal lacking under our laboratory conditions. Alternatively, it may be a specific trait of clinical isolates that would be more adapted to human infection, in which the T6SS would be “turned off” or its function affected by exopolysaccharide production, presence of antibiotic resistance genes or additional regulatory plasmids. In this context, while this manuscript was in revision, Weber and co-workers reported that the T6SS in some *A*. *baumannii* strains including clinical MDR isolates can be transcriptionally repressed by two plasmid-encoded TetR regulators [39]. Whole genome sequence analysis failed to identify sequence homologues of these regulators in the *A*. *baumannii* MDR clinical strains analysed in this work and consistently, we observed the presence of Hcp in total extracts of the corresponding cells thus suggesting a different T6SS regulatory mechanism at the level of secretion at play.

In contrast to the clinical strains, DSM30011 was able to strongly outcompete *E*. *coli* and more importantly other Gram-negative pathogens affecting the respiratory tract in a T6SS-dependent manner. This could be particularly relevant in the context of competition for a specific environmental niche but also in nosocomial infections, in which patients are often colonized by multiple bacterial pathogens, which could result in transfer of genetic material and spread of antibiotic resistance [14]. Genomic regions around *vgrG* loci were analysed in search for putative effectors in the DSM30011 strain that could account for its killing capacity. In the genomic region flanking DSM_11755 gene, which is the closest to the T6SS locus, no genes with function related to T6 apparatus were found. In contrast, in the other three *vgrG* loci (S5 Fig) genes encoding for candidate effectors only present in DSM30011 and not in the other strains under study were detected. Downstream the DSM_13300 gene, three ORFs with homology to Rhs proteins were found associated to a putative immunity protein (S5 Fig). Indeed, the ORF closest to the putative immunity protein contains a PxxxxDPxGL peptide motif (PNQWIDPKGL), which sharply demarcates the Rhs C-t region usually harboring a toxin [40]. No characterized domains were found in the C-t, which is conserved in Rhs proteins only encoded by the *Acinetobacter* genus. Close to DSM_18715 and DSM_00475 genes four putative proteins with peptidoglycan-binding domains were detected (PG1_1106, PG2_1106, LysM_932 and LysM_1106) two of them harbouring a LysM motif frequently present in accessory proteins of the T6SS [41]. PG2_1106 also shows homology to enzymes with muramidase activity. Of note, a protein harbouring a MIX (marker for T6 effector) domain and a pre-toxin motif was detected in the DSM_18715 loci. However, no known toxin domains were found in the C-t of this protein. In sum, several genes coding for toxin candidates were found in the analysed loci, which could be responsible for the capacity of DSM30011 to outcompete other bacteria.

In sharp contrast to ATCC17978 [10] we found that the DSM30011 T6SS also mediated virulence towards *G*. *mellonella*. The molecular mechanisms by which the T6SS modulates DSM30011 host colonization need to be further investigated. Our data suggest that it does not play a role in either bacterial adhesion to abiotic surfaces nor biofilm formation. Interaction with host cells, namely lung epithelial cells and phagocytic cells needs to be investigated in the context of the T6SS. It is possible that the T6SS would enable control of specific cellular responses or enhance resistance to killing by phagocytes, a cell type relevant in the *Galleria* model of infection. The innate immune systems of *G*. *mellonella* and mammals share a high degree of homology, and the larvae of this insect show both humoral responses with antimicrobial peptide production and basic cellular immune responses mediated mainly by phagocytic cells designated haemocytes [42]. This model organism has in fact been widely used for studying virulence of human pathogens such as *L*. *pneumophila* and *P*. *aeruginosa* with good correlation to mammalian models of infection [34–36].

In summary, we have shown that the *A*. *baumannii* DSM30011 T6SS is operative and critical for outcompeting other bacterial species. Importantly, we show for the first time in the case of this specific environmental *A*. *baumannii* strain, that the T6SS is also implicated in host colonisation. Additional studies are now needed to identify the secreted molecules involved, determine the role of the T6SS during the course of colonization and if it confers a fitness advantage in mixed infections *in vivo*, as is often the case for *A*. *baumannii* hospital infections.

## Supporting Information

S1 Fig Alignments of VgrG proteins of the *A*. *baumannii* strains analyzed in this study. The different VgrG proteins were predicted from nucleotide sequencing analysis conducted in this work and are indicated after their strain origin (Ab, Ab242/244/825 clinical strains; DSM, DSM30011; 17978, ATCC 17978) followed by the corresponding content of amino acids. The 17978_914* denomination denotes a hypothetical VgrG of 914 amino acid residues from ATCC 17978 which was inferred from the nucleotide sequence A1S_0082 after correcting for possible frameshift sequence errors on the basis of alignments with other VgrG sequences. The numbers above the sequences indicate the corresponding amino acid position used for comparisons in the text. The alignments were constructed by MUSCLE 3.7 with default parameters using the programs available in http://phylogeny.lirmm.fr. Similar residues are colored as the most conserved according to BLOSUM62 (average BLOSUM62 score: max: 3.0 (pale blue); mid: 1.5 (blue); low: 0.5 (gray). The domain of unknown function DUF2345/PF10106 (inferred using http://pfam.xfam.org) extends between residues 859–1004 in the alignments and contains deletions of different extents at its C-terminal region in VgrGs 17978_933 and Ab_788.
(PDF)

S2 Fig Hcp secretion under standard culture conditions. A) RT-PCR transcriptional analysis of *hcp* and *tssM* expression; *rpoB* gene expression was used as endogenous control. The RNA not subjected to RT was also run in PCR (bottom panel, negative control) to ensure that PCR positive reactions were due to the presence of transcripts and not contaminating genomic DNA. B) Detection of Hcp (arrows) in concentrated culture supernatants of the indicated *A*. *baumannii* strains grown up to exponential phase in TSB. Proteins were separated by 18% SDS-PAGE and stained by Coomasie Blue. Immunoblottings showing the presence of Hcp in whole cell lysates are shown at the bottom.
(TIFF)

S3 Fig Competition between *A*. *baumannii* species. Survival of the DSM30011 mini Tn7 (gentamicin-resistant; Gm^R^) strain after incubation in growth medium (control) or with wild-type or Δ*tssM A*. *baumannii* 17978 strains at a 10:1 ratio.
(TIFF)

S4 Fig Aggregate formation by DSM30011 strain. Confocal microscope images of biofilm formed on glass-bottom slides after 24 h in BM2G medium. Top image corresponds to 3D reconstruction to show large bacterial aggregates formed with the DSM30011 strain in contrast to the ATCC17978, with depth analysis below and corresponding surface plots. All scale bars correspond to 20 μm.
(TIFF)

S5 Fig Genetic organization of *vgrG* loci in *A*. *baumannii* DSM30011. Hyp, hypothetical protein; Rhs, Rhs homologous protein; Imm, putative toxin immunity protein; MIX, LysM and PG are MIX-, LysM- and peptidoglycan binding-domain containing proteins, respectively; Pre-tox, pre-toxin motif.
(TIFF)

S1 Table Strains and plasmid included in this study. (PDF)

S2 Table Primers used in this study (PDF)

S3 Table T6SS locus genes in *A*. *baumannii* strains under study. (PDF)

S4 Table Putative VrgG proteins encoded by the *A*. *baumannii* strains under study. (PDF)
