# Supplementary material for: Differential Role of the T6SS in Acinetobacter baumannii Virulence
Source: PLoS One. 2015 Sep 24;10(9):e0138265. doi: 10.1371/journal.pone.0138265 (PMC4581634; doi:10.1371/journal.pone.0138265)
Supplement: S1 Fig — The different VgrG proteins were predicted from nucleotide sequencing analysis conducted in this work and are indicated after their strain origin (Ab, Ab242/244/825 clinical strains; DSM, DSM30011; 17978, ATCC 17978) followed by the corresponding content of amino acids. The 17978_914* denomination denotes a hypothetical VgrG of 914 amino acid residues from ATCC 17978 which was inferred from the nucleotide sequence A1S_0082 after correcting for possible frameshift sequence errors on the basis of alignments with other VgrG sequences. The numbers above the sequences indicate the corresponding amino acid position used for comparisons in the text. The alignments were constructed by MUSCLE 3.7 with default parameters using the programs available in http://phylogeny.lirmm.fr. Similar residues are colored as the most conserved according to BLOSUM62 (average BLOSUM62 score: max: 3.0 (pale blue); mid: 1.5 (blue); low: 0.5 (gray). The domain of unknown function DUF2345/PF10106 (inferred using http://pfam.xfam.org) extends between residues 859–1004 in the alignments and contains deletions of different extents at its C-terminal region in VgrGs 17978_933 and Ab_788. (PDF) [file pone.0138265.s001.pdf]

A)

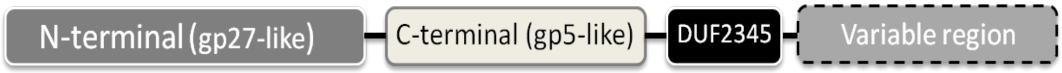

B)

```
            1                                                          60
Ab_1104     MlfNIFsVLEkIGLnaQkRAIHvQFSNelLNhQVFLQRIEGQHqLNgGLmAELICLSTNA
DSM_1106    MlfNIFsVLEkIGLnaQkRAIHvQFSNelLNhQVFLQRIEGQHqLNgGLmAELICLSTNA
17978_933   --------------------------------------------------mAELICLSTNA
Ab_1_463    MlnsIhQVLdSLGIspQkRAIrvQFtspiLNdQVFLQRIdGvHaLNdGLkAELlCLSTNA
Ab_2_601    ------------------------------------------------------------
17978_1_426 ------------------------------------------------------------
17978_914*  --msvssiLErLGLvSQnRAVHiQFSNqSLNqQVFLQRIEGeHtLNQGsvAELlCLSTNA
DSM_932     MqmsvssiLErLGLvSQnRAVHiQFSNqSLNqQVFLQRIEGeHtLNQGsgAELlCLSTNA
17978_2_395 ------------------------------------------------------------
DSM_875     MshkIFQiLdSLGLvaQnRvlHvQFSNASLNNQVFLQRIEGeHtLNQGsvAELlCLSTNA
Ab_1064     MfnNIFQiLESfGflSQHRsVyLQFSdASLNSQVFLQRIdGQHYLNQGMtAELICLSTNA
DSM_921     MfnNIsQVLESfGflSQHRsVyLQFSdASLNSQVFLQRIdGQHYLNQGMttELICLSTNA
Ab_788      MfnNIsQVLESLGflSQHRsVHLQFSdASLNSQVFLQRIEGQHYLNEGMtAELICLSTNA
17978_921   --------------------myLQFSdASLNSQVFLQRIdGQHYLNQGMtAELICLSTNA

            61                                                        120
Ab_1104     qIALKQFIGVQVAVDQVTDsGQlFRTTGIvTEASyGQSDGALTLYKLTIEDaTnLWHKRR
DSM_1106    qIALKQFIGVQVAVDQVTDsGQlFRTTGIvTEASyGQSDGALTLYKLTIEDaTnLWHKRR
17978_933   qIALKQFIGVQVAVDQVTDsGQlFRTTGIvTEASyGQSDGALTLYKLTIEDaTnLWHKRR
Ab_1_463    tIqLKsFIGVQaAVDiVTeRGeltRvTGIIThAqQGQSDGsLTLYKLTlEDPTaLWkyRR
Ab_2_601    ------------------------------------------------------------
17978_1_426 ------------------------------------------------------------
17978_914*  HIALKQFIGcQVAVDQVTDtGQFFRTTGIITEASQGQSDGsLTiYnLTlkDPTaLWHKRR
DSM_932     HIALKQFIGcQVAVDQVTDtGQFFRTTGIITEASQGQSDGsLTiYnLTlkDPTaLWHKRR
17978_2_395 ------------------------------------------------------------
DSM_875     HIALKQFIGcQVAiDQVTDtGQFFRTTGIITEASQGQSDGsLTiYnLTlkDPTaLWHKRR
Ab_1064     HIpLKtFIGVQVAVDQVTDRGsFFRTTGIITgASQGQSDGALTLYKLaIsDPTyLWHKRR
DSM_921     HIpLKtFIGlQVAVDQVTDRGsFFRTTGIITgASQGQSDGALTLYKLaIsDPTyLWHKRR
Ab_788      HIALKtFIGVQVAiDQVTDQGQlFRTTGIITgASQGQSDGALTLYKLTIsDPTyLWHKRR
17978_921   HIpLKtFIGlQVAVDQVTDRGsFFRTTGIITgASQGQSDGALTLYKLaIsDPTyLWHKRR

            121                                                       180
Ab_1104     NSRVFMNKSivEItEvLFKEWQeKSPLFAaSLsLDLgGLsQnYDiRPFtMQhNESDYDFL
DSM_1106    NSRVFMNKSivEItEvLFKEWQeKSPLFAaSLsLDLgGLsQnYDiRPFtMQhNESDYDFL
17978_933   NSRVFMNKSivEItEvLFKEWQeKSPLFAaSLsLDLgGLsQnYDiRPFtMQhNESDYDFL
Ab_1_463    NSRVFMNKSVvEIwEILFKEWQtKnPLFAaSLsLDLSGLtQTYDVRPFVMQhNESDwnFL
Ab_2_601    ------------------------------------------------------------
17978_1_426 -------------------------------------------------MQsNESDYDFL
17978_914*  NSRVFMNKSVRdISEILFKEWQGKSPLFASSiTLDtSGLtkdYDVRPFVMQsNESDYDFL
DSM_932     NSRVFMNKSVRdISEILFKEWQGKSPLFASSiTLDtSGLtkdYDVRPFVMQsNESDYDFL
17978_2_395 ------------------------------------------------------------
DSM_875     NSRVFMNKSVRdInEILFKEWQGKSPLFASSiTLDtSGLskdYDVRPFVMQsNESDYDFL
Ab_1064     NSRVFMNKSVKEISEILFqEWQGKSPLFASSLTLDLSGLKQTYDVRPFVMQlNESDYDFL
DSM_921     NSRVFMNKSVKEISEILFqEWQGKSPLFASSLTLDLSGLKQTYDVRPFVMQlNESDYDFL
Ab_788      NSRVFMNKSVREItEILFqEWQGKSPLFASSLTLDLSGLKQaYDiRPFVMQlNqSDYDFL
17978_921   NSRVFMNKSVKEISEILFqEWQGKSPLFASSLTLDLSGLKQTYDVRPFVMQlNESDYDFL

            181                                                       240
Ab_1104     TRLWRSEGvSWLIDESELFVphfTApIQaQKLRLIDDNNQYQALARRSIRYHRSSATEyq
DSM_1106    TRLWRSEGvSWLIDESELFVphfTApIQaQKLRLlDDNNQYQALARRSIRYHRSSATEyq
17978_933   TRLWRSEGvSWLIDESELFVphfTApIQgQKLRLIDDNNQYQALARRSIRYHRSSATEyq
Ab_1_463    TRLlRSEnISWLIDEaqhiVpStdtSIQaQKLRLIDaNsQYQpLdRktIRYHRSSAvEQY
Ab_2_601    ------------------------------------------------------------
17978_1_426 TRLWRSEGInWLvDESqLFVAdpnASIQPQvLRLIDDNqnYQALeRRSIRYqRSSATEQF
17978_914*  TRLWRSEGInWLvDESqLFVAdpnASIQPQvLRfnrpkfirhlnAvaSaitvsSatravp
DSM_932     TRLWRSEGInWLvDESqLFVAdpnASIQPQvLRLIDDNqnYQALeRRSIRYqRSSATEQF
17978_2_395 ------------------------------------------------------------
DSM_875     TRLWRSEGInWLvDESqLFVAdpnASIQPQvLRLIDDNqnYQALeRRSIRYqRSSATEQF
Ab_1064     TRLWRSEGISWLIDEaELtVASnmdNIQPQKLRLIDDNNQYQALtRRaIRYHRSSATEQF
```

```
DSM_921         TRLWRSEGISWLIDEaELtVASnmdNIQPQKLRLIDDNNQYQALtRRaIRYHRSSATEQF
Ab_788          TRLWRSEGInWLIDEaELtVpSnTdhIQPQKLRLIDDNsQYQALSRRtIRYHRShATEQF
17978_921       TRLWRSEGISWLIDEaELtVASnTdNIQPQKLRLIDDNsQYQtLtRRtIRYHRSSATEQF

             241                                                        300
Ab_1104         DSITgfvAvRSLQPTaVHVQRWQpDALaQEEGNGSVvtTHtHSDNfDsAtLSLEqAWHVS
DSM_1106        DSITgfvAvRSLQPTaVHVQRWQpDALaQEEGNGSVvtTHtHSDNfDsAtLSLEqAWHVS
17978_933       DSITgfvAvRSLQPTaVHVQRWQpDALaQEEGNGSVvtTHtHSDNfDsAtLSLEqAWHVS
Ab_1_463        DSmTRLtAeRSLQPnvmHiQRWQAeiLdQEEGiGSVQSkHqHSEhYDNAtLgLEqAWnyS
Ab_2_601        ------------------------------------------------------------
17978_1_426     DtITQvKAeRrLQPTSVHVQRWQADALQQEEGSGSVQgTqKHSEhYDNASLnLEDAWHVS
17978_914*      irITQvKAeRrLQPTSVHVQRWQADALQQEEGSGSVQgTqKHSEhYDNASLnLEDAWHVS
DSM_932         DtITQvKAeRrLQPTSVHVQRWQADALQQEEGSGSVQgTqKHSEhYDNASLnLEDAWHVS
17978_2_395     ------------------------------------------------------------
DSM_875         DtITQvKAeRrLQPTSVHVQRWQADALQQEEGSGSVQgTqKHSEhYDNASLnLEDAWHVS
Ab_1064         DSmTsLmAdRSLQPTSifVQRWQpDvLQQtdGAGSVQSkHqHStNYDNqSLSLEeAWHfS
DSM_921         DSmTsLmAdRSLQPTSifVQRWQpDvLQQtdGAGSVQSkHqHStNYDNqSLSLEeAWHfS
Ab_788          DSmTsLmAnRSLQPTSVfVQRWQsDvLQQtdGAGSVQSTHqHStNYDNqSLSLEDAWHfS
17978_921       DSmTsLiAgRhLQPsSVfVQRWQsDvLQQtdGAGSVQSkHeHSsNYDNqSLnLEDAWHfS

             301                                                        360
Ab_1104         PAWMQDLkGEDQATASSsSQLEKLNQHftdmHaSraKyFkAyssVRDTQVGYWFnLrEHP
DSM_1106        PAWMQDLkGEDQATASSsSQLEKLNQHftdmHaSraKyFkAyssVRDTQVGYWFnLrEHP
17978_933       PAWMQDLkGEDQATASSsSQLEKLNQHftdmHaSraKyFkAyssVRDTQVGYWFnLrEHP
Ab_1_463        PAWigDLkGEDgvTkSgNqQvErLNQnLhnYyEAQaKrFiAqTTVRDayVGYyFELNEHP
Ab_2_601        ------------------------------------------------------------
17978_1_426     PAWMQDLNGkDQATASgNSQiEqLNQHINAYHhlsSKQFtvagnVRDaQVGYWFELNdHP
17978_914*      PAWMQDLNGEDQATASgNSQiEqLNQHINAYHhlsSKQFtvagnVRDaQVGYWFELNdHP
DSM_932         PAWMQDLNGEDQATASgNSQiEqLNQHINAYHhlsSKQFtvagnVRDaQVGYWFELNdHP
17978_2_395     ------------------------------------------------------------
DSM_875         PAWMQDLNGEDQATASgNSQiEqLNQHINAYHhlsSKQFtvagnVRDaQVGYWFELNdHP
Ab_1064         PAWMQDLNGEDgATsaSNqQiEKfNQnLsAYyDAQSKQFiAkTTVRDTQVGYWFELNEHP
DSM_921         PAWMQDLNGEDgATsaSNqQLEKfNQnLsAYyDAQSKQFiAkTTVRDTQVGYWFELNEHP
Ab_788          PAWMQDLkGEDgATsaSNqQLEKfNQnLsAfHDAQSKQFiAqTTVRDTQVGYWFELNEHP
17978_921       PAWMQDLNGEDgATsaSNqQLEKfNQnLsAYHDAQSKQFvAqTTVRDTQVGYWFELNEHP

             361                                                        420
Ab_1104         EIDQHEGADqEFLIIaKnfYNQNNLPKDLhQQVsQLLTQ-----SrWDqhgydDI-ERQG
DSM_1106        EIDQHEGADqEFLIIaKnfYNQNNLPKDLQQQVsQLLTQ-----SrWDqhgydDI-ERQs
17978_933       EIDQHEGADqEFLIIaKnfYNQNNLPKDLhQQVsQLLTQ-----SrWDqhgydDI-ERQG
Ab_1_463        EIDQHEssDKsFLIVSKnffNQNNLPKDLndQIngLLaQ-----SnWaiQNpEnsdERQa
Ab_2_601        ------------------------------------------------------------
17978_1_426     ElDQHdsADKEFLIlSKHYYNQNNLPKeLQQQlERLLpQGKLKAaQlDSQNpE---qRhf
17978_914*      ElDQHdsADKEFLIlSKHYYNQNNLPKeLQQQlERLLpQGKLKAaQlDSQNpE---qRhf
DSM_932         ElDQHdsADKEFLIlSKHYYNQNNLPKeLQQQlERLLpQGKLKAaQlDSQNpE---qRhf
17978_2_395     ------------------------------------------------------------
DSM_875         ElDQHdsADKEFLIlSKHYYNQNNLPKeLQQQlERLLpQGKLKAaQlDSQNpE---qRhf
Ab_1064         EIDQHEstDKEFLIIgKnYYNQNNLPKDLnQQIQnLvqQ-----SdWqasNtd---ERQa
DSM_921         EIDlHsGADKEFLIIgKsYYNQNNLPKDLdQQIQtLtTQ-----ShWqSshtk---ERQG
Ab_788          EIDQHnGsDKEFLIIgKHYYNQNNLPKDLnQQIQaLvrQ-----ShW-SiNnE---ERQG
17978_921       EIDlHsGADKEFLIIgKHYYNQNNLPKDLdQQIQtLtTQ-----ShWqSshtk---ERQG

             421                                                        480
Ab_1104         NELtLiRRqIKTaPEYNPeQHRPiAyPQRAkVVGPEGEtIHVDeWGRIKVRFLFTRSDDH
DSM_1106        NELtLiRRqIKTaPEYNPeQHRPiAyPQRAkVVGPEGEtIHVDeWGRIKVRFLFTRSDDH
17978_933       NELtLiRRqIKTaPEYNPeQHRPiAyPQRAkVVGPEGEtIHVDeWGRIKVRFLFTRSDDH
Ab_1_463        NqLiLqRRHIpTtPaYNPqiHsPvtHPQRAkVVGPEGEeIyVDeWGay------------
Ab_2_601        -------------------------------------msGGRIKVRFLFTRSDDH
17978_1_426     aELnvvRRnIKaVPEYNPlEHRPaAHPQRARVVGlEGESIHVDQWGRIKVRFL-------
17978_914*      c--aecRsshqscailirstdqPltHsa-pRVVGlEGESIHVDpvGR-hsRFLFTRaDDH
DSM_932         aELnvvRRnIKaVPEYNPlEHRPaAHPQRARVVGlEGESIHVDQWGRIKVRFLFTRtDDH
17978_2_395     ------------------------------------------------------------
DSM_875         aELnvvRRnIKaVPEYNPlEHRPaAHPQRARVVGlEGESIHVDQWGRIKVRFLFTRaDDH
Ab_1064         NqLiLqRRHIpTtPaYNPqiHsPvtHPQRAkVVGPEGEeIyVDeWGRIKVRFLFTRSDDH
DSM_921         NqLiLqRRHIKTVPEYqPlQdRPqAsvQRARVVGPEGESIHVDQWGRIKVRFLFTRSnDH
Ab_788          NELiLqRRHIKTVPEYqPlQdRPqAsvQRARVVGPEGESIHVDQWGRIKVRFLFTRaDDH
17978_921       NqLiLqRRHIKTVPEYqPlQdRPqAsvQRARVVGPEGESIyVDQWGRIKVRFLFTRanDH

             481                                                        540
Ab_1104         gHDGGAGSNDNDTDSAWVDVLTPWAGEGYGARFLPRIGEVVVIDFFDGNIDRPFVtGRIH
DSM_1106        gHDGGAGSNDNDTDSAWVDVLTPWAGEGYGARFLPRIGEVVVIDFFDGNIDRPFVtGRIH
```

```
17978_933    gHDGGAGSNDNDTDSAWVDVLTPWAGEGYGARFLPRIGEVVVIDFFDGNIDRPFVtGRIH
Ab_1_463     ------------------------------------------------------------
Ab_2_601     SHDGGAGtNnNDTDSAWiDVLTPWAGEGYGARFLPRIGEIVVIDFFnGdIDRPFVmGRIH
17978_1_426  ---------------------------------LPelmttammvelevttmtPtrlGsmy
17978_914*   SHDGGAGSNDNDTDSAWVDVLTPWAGaGYGARFLPRvGEIVVIDFFDGNIDRPFVVGRIH
DSM_932      SHDGGAGSNDNDTDSAWVDVLTPWAGaGYGARFLPRvGEIVVIDFFDGNIDRPFVVGRIH
17978_2_395  ------------------------------------------------------mVGRIH
DSM_875      SHDGGAGSNDNDTDSAWVDVLTPWAGaGYGARFLPRvGEIVVIDFFDGNIDRPFVVGRIH
Ab_1064      SHDGGAGtNnNDTDSAWiDVLTPWAGEGYGARFLPRIGEIVVIDFFnGdIDRPFVmGRIH
DSM_921      SHDGGAGSNDNDTDSAWVDVvTPWAGkGYGARFLPRvGEIVVIDFFDGNIDRPFVVGRIH
Ab_788       rHDGGAGSNDNDTDSAWVDVLTPWAGEGYGARFLPRIGEIVVIDFFDGNvDRPFVVGRIH
17978_921    SHDGGAGSNDNDTDSAWVDVLTPWAGaGYGARFLPRvGEIVVIDFFDGNIDRPFVVGRIH

          541                                                         600
Ab_1104      EAqRsPTkFDvKGQLPDTKKLSGIRSqEinGsGFNQLRFDDTTGQIStQLQSSHAAtQLN
DSM_1106     EAqRsPTkFDvKGQLPaTKKLSGIRSqEinGsGFNQLRFDDTTGQIStQLQSSHAAtQLN
17978_933    EAqRsPTkFDvKGQLPDTKKLSGIRSqEinGsGFNQLRFDDTTGQIStQLQSSHAAtQLN
Ab_1_463     ------------------------------------------------------------
Ab_2_601     EAqRqPTkFDnKGkLPDTKKLSGIRSkEVsGgGFgQLRFDDTpGQIStQLQSSHgASQLN
17978_1_426  s-------llgqGQv-----------------------mahvschvlvkLlSSisltvml
17978_914*   EAERHPTQFDQKGQLPDTKKLSGIRSEEVDGKGFNQLRFDDTTGQISAQLQSSHAASQLN
DSM_932      EAERHPTQFDQKGQLPDTKKLSGIRSEEVDGKGFNQLRFDDTTGQISAQLQSSHAASQLN
17978_2_395  EAERqPTQFDQKGQLPDTKKLSGIRSEEVDGKGFNQLRFDDTTGQISAQLQSSHAASQLN
DSM_875      EAERHPTQFDQKGQLPDTKKLSGIRSEEVDGKGFNQLRFDDTTGQISAQLQSSHAASQLN
Ab_1064      EAqRHPTkFDnKGkLPDTKKLSGIRSkEVsGgGFgQLRFDDTpGQIStQLQSSHgASQLN
DSM_921      EAERHPaQFDQKGQLPDTKKLSGIRSEEVDGKGFNQLRFDDTTGQISAQLhSSHAASQLN
Ab_788       EAERHPTQFDQKGQLPDTKKLSGIRSEEVDGKGFNQLRFDDTTGQISAQLQSSHAASQLN
17978_921    EAERHPaQFDQKGQLPDTKKLSGIRSEEVDGKGFNQLRFDDTTGQISAQLQSSHAvSQLN

          601                                                         660
Ab_1104      LGNLSHPKeqAtSqGRGEGFELRTDaWGA-VRAGKGmLISTyaQeQAiadHLeAAQAqsl
DSM_1106     LGNLSHPKeqemSqGRGEGFELRTDaWGA-VRAGKGmLISTyaQeQAiadHLeAAQAqsl
17978_933    LGNLSHPKeqetSqGRGEGFELRTDaWGA-VRAGKGmLISTyaQeQAiadHLeAAQAqsl
Ab_1_463     ------------------------------------------------------------
Ab_2_601     LGkLSHPKDKAESedRGEGFELRTDQWGA-lRAGqGLLVSTHKQDnAKGdHLDAevAKkQ
17978_1_426  tarLw---------------------WGvfmkpsanqrsSTrKasyltprnLavSvqKks
17978_914*   LGNLSHPKDKAESDGRGEGFELRTDQWGA-VRAGsGLLVSTHKQDQAQGvHLDASEAKQQ
DSM_932      LGNLSHPKDKAESDGRGEGFELRTDQWGA-VRAGsGLLVSTHKQDQAQGvHLDASEAKQQ
17978_2_395  LGNLSHPKDKAESDGRGEGFELRTDQWGA-VRAGsGLLISTHKQDQAQGvHLDASEAKQQ
DSM_875      LGNLSHPKDKAESDGRGEGFELRTDQWGA-VRAGsGLLVSTHKQDQAQGvHLDASEAKQQ
Ab_1064      LGkLSHPKDKAESedRGEGFELRTDQWGA-lRAGqGLLVSTHKQDnAKGdHLDAgvAKkQ
DSM_921      LGNLSHPKDKAESDGRGEGFELRTDQWGA-VRAGKGLLVSTHKQDQAQGvHLDANnAeQQ
Ab_788       LGNLSHPKeKAESDGRGEGFELRTDQWGA-VRAGsGLyVSTHKQDQAQGtHLDAkEtKQQ
17978_921    LGNLSHPKDKAESDGRGEGFELRTDQWGA-VRAGKGLLVSTHKQDQAQGtHLDAADAKQQ

          661                                                         720
Ab_1104      lsqGyesmKmLSEVAakQQTDaLNVINRLPKFIQSLELKTTGQALNSTVNLFKEGINNDP
DSM_1106     lsqGyesmKmLSEVAakQQTDaLNVINRLPKFIQSLELKTTGQALNSTVNLFKEGINNDP
17978_933    lsqGyesmKmLSEVAakQQTDaLNVINRLPKFIQSLELKTTGQALNSTVNLFKEGINNDP
Ab_1_463     ------------------------------------------------------------
Ab_2_601     lEGsqtNsKALSdiAKNQkTDei-------------------------------------
17978_1_426  makaLtss----------------------------------------------------
17978_914*   IEGGLNNAKALSEVAKNQQTDPL-------------------------------------
DSM_932      IEGGLNNAKALSEVAKNQQTDPL-------------------------------------
17978_2_395  IEGGLNNAKALSEVAKNQQTDPL-------------------------------------
DSM_875      IEGGLNNAKALSEVAKNQQTDPL-------------------------------------
Ab_1064      IEGsqtNsKALSdiAKNQkTDei-------------------------------------
DSM_921      IEGsLNNAKALSEVAKNQQTDPL-------------------------------------
Ab_788       ldGnLNNAKALSdVAKNQQTDPL-------------------------------------
17978_921    IEGGLNNAKALSEVAKNQQTDPL-------------------------------------

          721                                                         780
Ab_1104      IHALKDCGGFIQDIGALGGNAKGVVDEFNSFFSDAKdavENLKaFIEnVEEHgPDIVKGK
DSM_1106     IHALKDCGGFIQDIGALGGNAKGVVDEFNSFFSDAKdavENLKaFIEnVEEHgPDIVKGK
17978_933    IHALKDCGGFIQDIGALGGNAKGVVDEFNSFFSDAKdavENLKaFIEnVEEHgPDIVKGK
Ab_1_463     ------------------------------------------------------------
Ab_2_601     ------------------------------------EsiEqLKdFasQIqq---------
17978_1_426  ------------------------------------------------------------
17978_914*   ------------------------------------EMLENLKTFIEQIEEkDqD-----
DSM_932      ------------------------------------EMLENLKTFIEQIEEkDqD-----
17978_2_395  ------------------------------------dMLENiqTFLEvlkqeDPk-----
```

```
DSM_875          ------------------------------------dMLENiqTFLEvlkqeDPk-----
Ab_1064          ------------------------------------EsiEqLKdFasQIqq---------
DSM_921          ------------------------------------EVLENLKnFLEQIEqqDkD-----
Ab_788           ------------------------------------EhLENLKTFLEQIEEqDqg-----
17978_921        ------------------------------------EMLENLKTFIEQIEEkDqD-----

               781                                                        840
Ab_1104          LASIKDRIHKNPFESIQEVGrVLANVETKDFDLMSTCGTFSKGSKLEVSPSKALSSLQGF
DSM_1106         LASIKDRIHKNPFESIQEVGKVLANVETKDFDLMSmCGTFSKGSKLEVSPSKALSSLQGF
17978_933        LASIKDRIHKNPFESIQEVGKVLANVETKDFDLMSTCGTFSKGSKLEVSPSKALSSLQGF
Ab_1_463         ------------------------------------------------------------
Ab_2_601         ------------------------------------------------------------
17978_1_426      ------------------------------------------------------------
17978_914*       ------------------------------------------------------------
DSM_932          ------------------------------------------------------------
17978_2_395      ------------------------------------------------------------
DSM_875          ------------------------------------------------------------
Ab_1064          ------------------------------------------------------------
DSM_921          ------------------------------------------------------------
Ab_788           ------------------------------------------------------------
17978_921        ------------------------------------------------------------

               841                                                        900
Ab_1104          MEGYTQGLESSSDTKQQEqgkiFrQALMLLASPNgIALttpEnIiLqAsqdiaeSAsgSI
DSM_1106         MEGYTQGLESSSDTqQQEqgkiFrQALMLLASPNgIALttpEnIiLqAsqdiaeSAsgSI
17978_933        MEGYTQGLESSSDTKQQEqgkiFrQALMLLASPNgIALttpEnIiLqAsqdiaeSAsgSI
Ab_1_463         ------------------------------------------------------------
Ab_2_601         ------------------qiAkFekALlLLsSPdgIALsSsEDIHiSADaQiNQiAGDSI
17978_1_426      ----------------------------vLmtPqaksvpSy-------------------
17978_914*       ------------------KAAaFKQALMiLtaPNSIALASNEDIHLSADGQLNQtAGDSI
DSM_932          ------------------KAAaFKQALMiLtaPNSIALASNEDIHLSADGQLNQtAGDSI
17978_2_395      ------------------KAAeFqsAvMLLASPkSIAvsSNEDIHLSAnGQLtQSAGDSI
DSM_875          ------------------KAAeFqsAvMLLASPkSIAvsSNEDIHLSAnGQLtQSAGDSI
Ab_1064          ------------------qiAkFekALlLLsSPdgIALsSsEDIHiSADaQiNQiAGDSI
DSM_921          ------------------KAAvFKQAiMvLsaPNSVAiASNEDIHLSADGQLsQSAGDSI
Ab_788           ------------------KAAaFKQALMvLtaPNSIALASNEDIHLSADGQLsQtAGDSI
17978_921        ------------------KAAtFKQALMvLtaPNSIAvASNEDIHLSADGQLsQnAGDSI

               901                                                        960
Ab_1104          NLSaQKNIIgHAQdKISLFAAQkGlsafAAKGpIkvQAQteGieIlsRKnIkILSvEDKI
DSM_1106         NLSaQKNIIgHAQdKISLFAAQkGlRaYAAKGKlElQAQdDaieaIAkKvIklISTEDKI
17978_933        NLSaQKNIIgHAQdKISLFAAQkGlRaYAAKGKlElQAQdDaieaIAkKvIklISTEDKI
Ab_1_463         ------------------------------------------------------------
Ab_2_601         NiSTQKNVIAHAQNrlSLFAAQsGlkavAAqGKVEIQAQsDalDvlAnKGItIsSTEDcI
17978_1_426      --------------KvampqAs--------------------------------------
17978_914*       NLSTQKNlIAHAQNKISLFAAQQGARLYAgKGKVEIQAQGDGADLIARKavQvISTEDKI
DSM_932          NLSTQKNlIAHAQNKISLFAAQQGARLYAgKGKVEIQAQGDGADLIARKavQvISTEDKI
17978_2_395      NmSTQKNIvnHAsqKISLFAAQEGARLfAgKGKVEIQAQGDGlDvIARKGIQItSTEDtv
DSM_875          NmSTQKNIvnHAsqKISLFAAQEGARLfAgKGKVEIQAQGDGlDvIARKGvQItSTEDtv
Ab_1064          NiSTQKNVIAHAQNrlSLFAAQsGlkavAAqGKVEIQAQaDalDvlsklGItIsSTdDKv
DSM_921          NLSTQKNIIAHAQNKISLFAAQQGARLYAgnGKVEIQAQaDGADLIARKGvQIISTEDRI
Ab_788           sLSTQKNlIAHAQNKISLFAAQEGARLYAAKGKlEvQAQGe-------------------
17978_921        sLSTQKNlIAHAQNKISLFAAQQGARLYAgKGKVEIQAQGDGADLIARKGIQIISTEDKI

                961                                                      1020
Ab_1104          EIvgqKEIVLnAGGSQltIsdkGvFinTprlFhaKAGQHkFdaGAiINysfPnLPs----
DSM_1106         ELTSPKEIVLTAGGSQlKINanGvFstTGGKFEsKAGQHLFtsGAtVNaelPkMPe---t
17978_933        ELTSPKEIVLTAGGSQlKINanGvF-----------------------------------
Ab_1_463         ------------------------------------------------------------
Ab_2_601         EIsSPKEIViTgasSQItlNGSGIFPkTGGKFqVnAGQHvFqGGASasvqsslpPp-pKr
17978_1_426      ------------------------------------------------------------
17978_914*       EaTaaKEIVLTAGGSQVKItGSGIFmtTsGKFEVKAGQHLFMGGgSVNsslPyLPe-qgk
DSM_932          EaTaaKEIVLTAGGSQVKItGSGIFmtTsGKFEVKAGQHLFMGGgSVNsslPqLPe--ae
17978_2_395      fITSPtEInLTAnGSQVKlNGSGIFPVTGGKlEVKAGQHLFMGGeqVt-----pPkkqEn
DSM_875          yITSPtEInLTAnGSQVKlNGSGIFPVTGGKlEVKAGQHLFMGGsSINppaldLP-----
Ab_1064          iIsSPKEvkiTgGsSQItlNGSGIFPkTGGKFqVnAGQHLFMaGASaNagaPeLPk-aKp
DSM_921          EITasKEvViTsGGSQIKINsSGIFPVTGGKFEVKAGQHLFtsGASVsantPqLPvlgEl
Ab_788           -----KslIvsf-------------------------QrLykG-----------------
17978_921        EInasKEIViTsGGSQIKINsSGIFPVTGGKFEVKAGQHvFMGGAkadyvlPsLPt-qEi

              1021                                                       1080
```

```
Ab_1104      -myygnFnitDknnnpig-GqKYkmtlp-sGKeilGftDeNGntvtgysgednqnLklEi
DSM_1106     gmysMrFdlsqifdtkilknmEYklinhskkieavyefeqessARVysdsvdnveLal--
17978_933    ------------------------------------------------------------
Ab_1_463     ------------------------------------------------------------
Ab_2_601     vqgvLelfheyahGefvK-GgsYrVvdn-fGKevtGkLDdkGfAKVsglatGavKvfFEs
17978_1_426  ------------------------------------------------------------
17978_914*   qkygvwFdvmDkqGnklKpGtEYiIFdehdkEierGkLDrtGlvKL--------------
DSM_932      npfvLqYlvkNkenqvms-ekpYilldd-eGnihrGttDkdGfmKL--------------
17978_2_395  kpcyLtFeitDldGkpaK-nvEYiaFrm-dGsrqkGqtnaqGltQr--------------
DSM_875      ----------------------------dcsakqtqaaqNGsAKVdls------------
Ab_1064      mqgaLellrsyggdnfFK-qnsYkVids-lGKqitGkLDgNGfAQVtgiapGpAKvvFEk
DSM_921      nqhnLrYllkDkeGipFa-hhKYiaFmp-nGKkleGitDeNGytQL--------------
Ab_788       ------------------------------------------------------------
17978_921    ktddLlleylhsdGtpvK-GaDYeVlls-dGsirkGkLDasGkAiVsgvpaGrAKiqYge

         1081                                                        1140
Ab_1104      ----------------------------------------iedlyqdiwyqpnstyEyet
DSM_1106     ----------------------------------------vpgvylteikeliseqEaes
17978_933    ---------------------------------------------dnrw-----------
Ab_1_463     ------------------------------------------------------------
Ab_2_601     DHrdpWdtASDFKRpveWpnkndAdseqsdSlIaqMSKtaqsklgelskqltnptnimkn
17978_1_426  ------------------------------------------------------------
17978_914*   -----------------------------------------------eteepnkqyKihv
DSM_932      ----------------------------------------kttsasqrittrvmmnEieq
17978_2_395  ----------------------------------------fetdgseqisihicdenask
DSM_875      ------------------------------------------------------------
Ab_1064      DNtsaWlqSSDFKRnytWaepvkSvqglmkNaLeaVgqntmsqlqnnllstdknsfKnlg
DSM_921      ----------------------------------------iktvrpeeisihlynnEein
Ab_788       ------------------------------------------------------------
17978_921    Dq----------------------------------SKdefpalevddwftqlgsstktg

         1141                                                        1200
Ab_1104      iDdlElpvnfdkEvdEde------------------------------------------
DSM_1106     lDdeDidscgcgEehEhd------------------------------------------
17978_933    ------------------------------------------------------------
Ab_1_463     ------------------------------------------------------------
Ab_2_601     iqtaqsiksegaKalmpmLktqAqglVTdQVksfLpisEagqkignSielTsiqKMndfn
17978_1_426  ------------------------------------------------------------
17978_914*   vn----------------------------------------------------------
DSM_932      aDeeEagtee--------------------------------------------------
17978_2_395  yklaakg-----------------------------------------------------
DSM_875      ------------------------------------------------------------
Ab_1064      kntlDnlagqtvaqiKnqVtntAlntVSkQLnlnLsadQmkslgqmAtnpSqslEMlkeq
DSM_921      iD----------------------------------------------------------
Ab_788       ------------------------------------------------------------
17978_921    kEe---------------------------------------------------------

         1201
Ab_1104      ------------------------------------
DSM_1106     ------------------------------------
17978_933    ------------------------------------
Ab_1_463     ------------------------------------
Ab_2_601     ksgsIdgnslvnnTlhqylqSPfKKnS---------
17978_1_426  ------------------------------------
17978_914*   ------------------------------------
DSM_932      ------------------------------------
17978_2_395  ------------------------------------
DSM_875      ------------------------------------
Ab_1064      ggdfLsdqmtaklSkstnqdSPiQQgAldtfvrskk
DSM_921      ------------------------------------
Ab_788       ------------------------------------
17978_921    ------------------------------------
```

S1 Fig.
