# Supplementary figures and images for: Differential Role of the T6SS in Acinetobacter baumannii Virulence
Source: PLoS One. 2015 Sep 24;10(9):e0138265. doi: 10.1371/journal.pone.0138265 (PMC4581634; doi:10.1371/journal.pone.0138265)

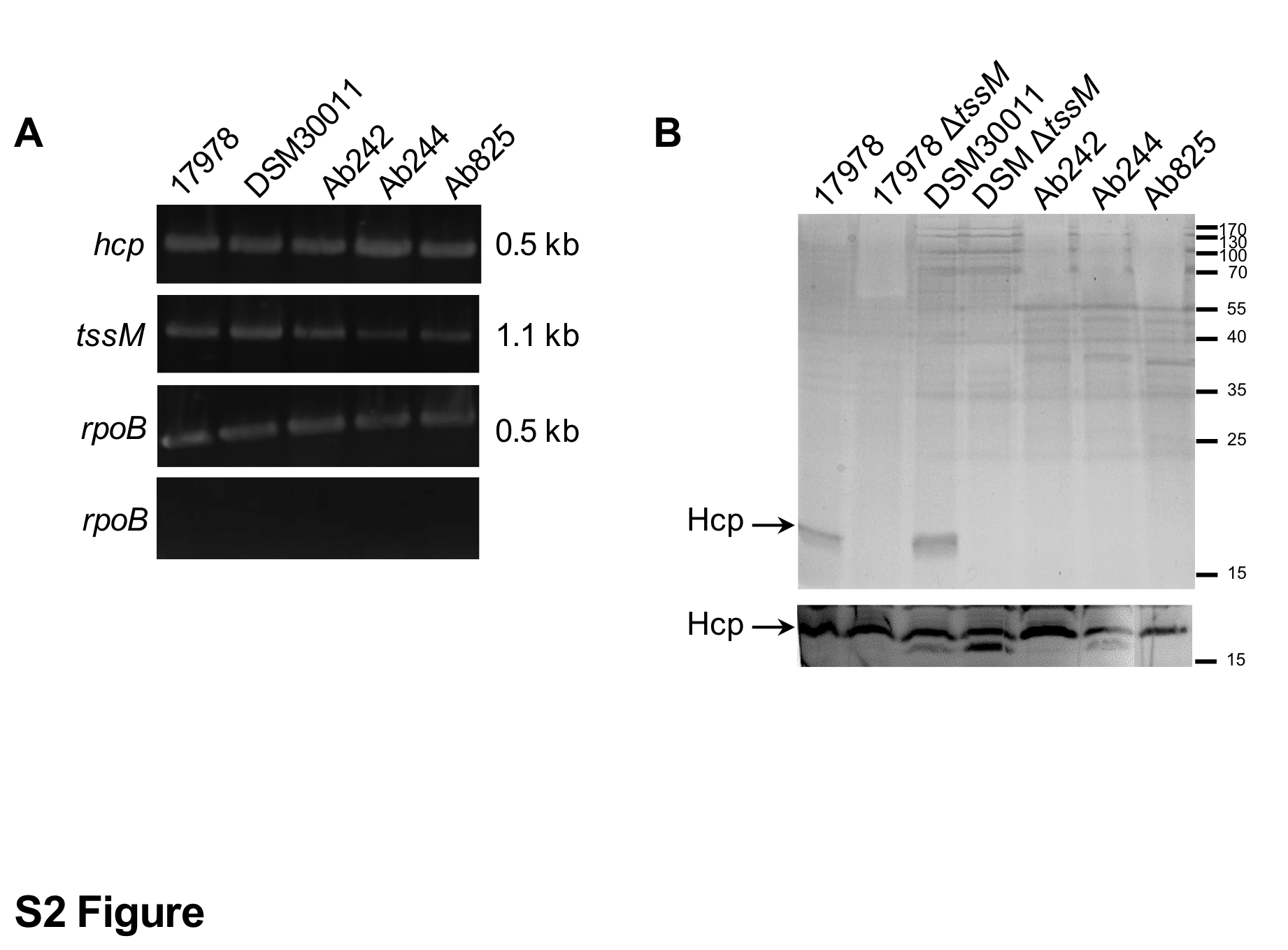

Supplement: S2 Fig — A) RT-PCR transcriptional analysis of hcp and tssM expression; rpoB gene expression was used as endogenous control. The RNA not subjected to RT was also run in PCR (bottom panel, negative control) to ensure that PCR positive reactions were due to the presence of transcripts and not contaminating genomic DNA. B) Detection of Hcp (arrows) in concentrated culture supernatants of the indicated A. baumannii strains grown up to exponential phase in TSB. Proteins were separated by 18% SDS-PAGE and stained by Coomasie Blue. Immunoblottings showing the presence of Hcp in whole cell lysates are shown at the bottom. (TIFF) [file pone.0138265.s002.tiff]

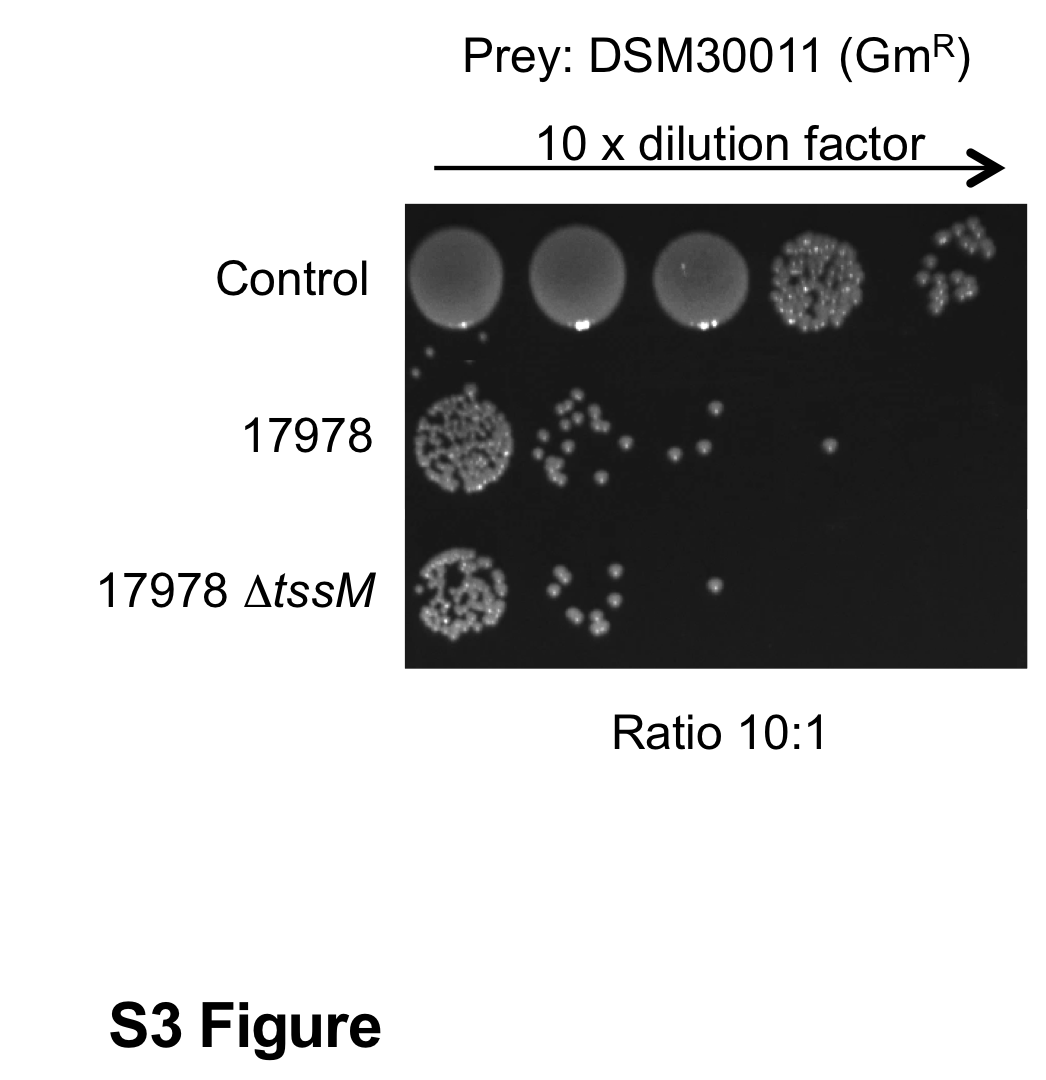

Supplement: S3 Fig — Survival of the DSM30011 mini Tn7 (gentamicin-resistant; GmR) strain after incubation in growth medium (control) or with wild-type or ΔtssM A. baumannii 17978 strains at a 10:1 ratio. (TIFF) [file pone.0138265.s003.tiff]

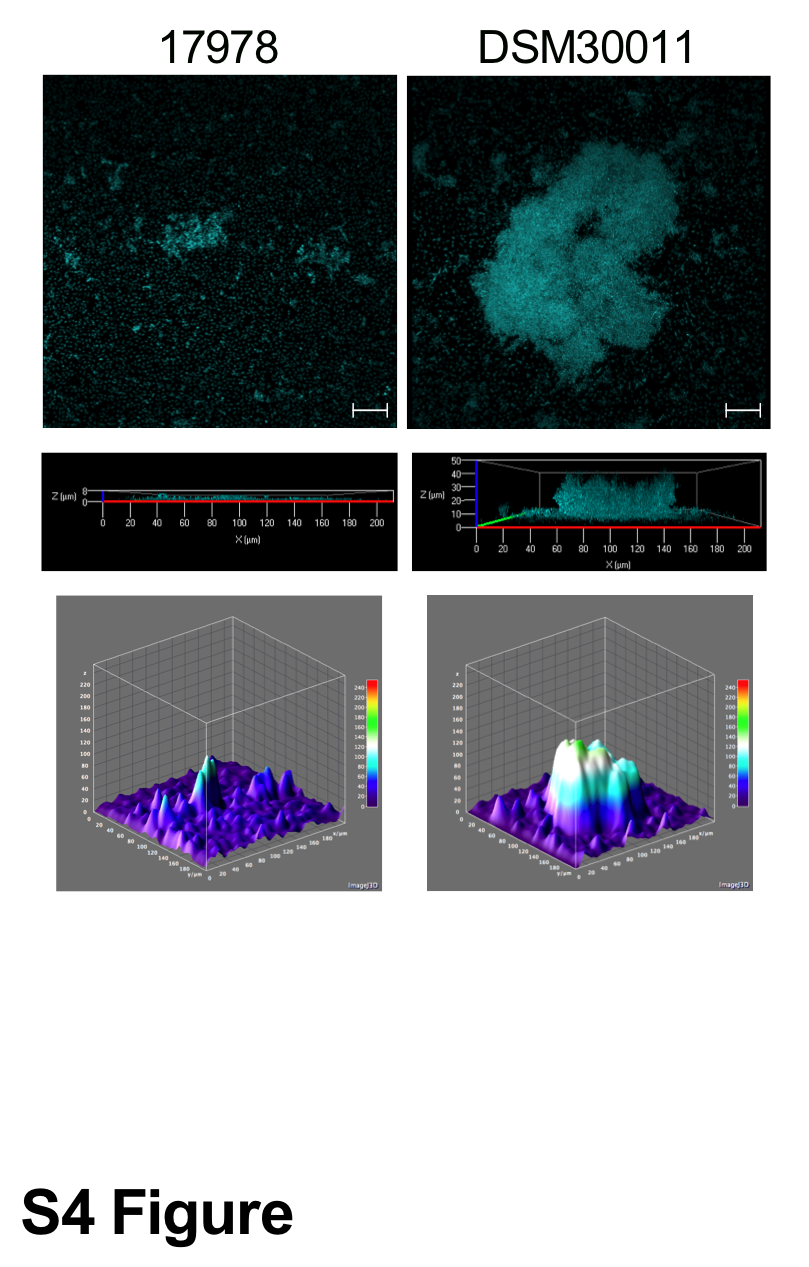

Supplement: S4 Fig — Confocal microscope images of biofilm formed on glass-bottom slides after 24 h in BM2G medium. Top image corresponds to 3D reconstruction to show large bacterial aggregates formed with the DSM30011 strain in contrast to the ATCC17978, with depth analysis below and corresponding surface plots. All scale bars correspond to 20 μm. (TIFF) [file pone.0138265.s004.tiff]

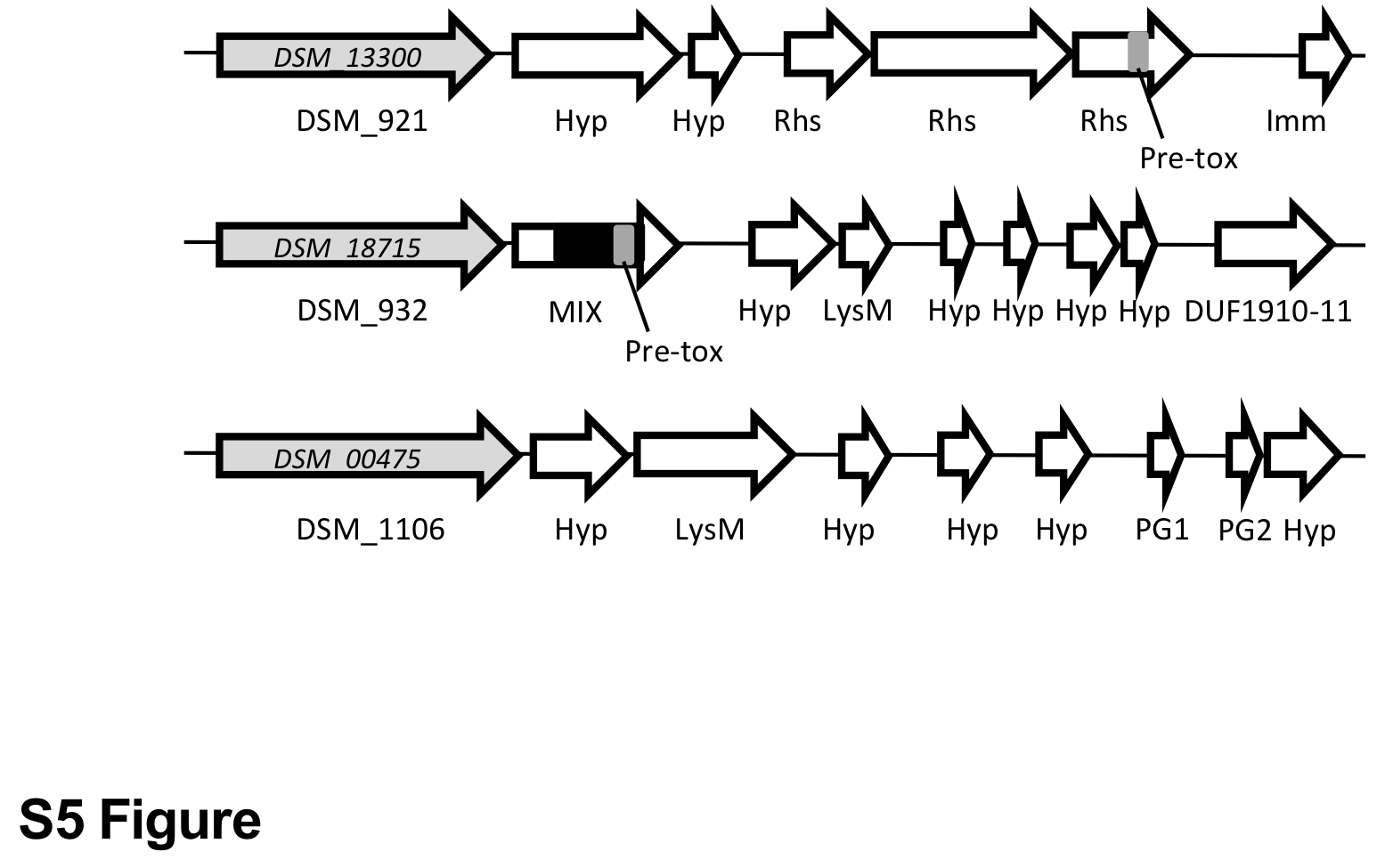

Supplement: S5 Fig — Hyp, hypothetical protein; Rhs, Rhs homologous protein; Imm, putative toxin immunity protein; MIX, LysM and PG are MIX-, LysM- and peptidoglycan binding-domain containing proteins, respectively; Pre-tox, pre-toxin motif. (TIFF) [file pone.0138265.s005.tiff]
