# Supplementary material for: Differential Role of the T6SS in Acinetobacter baumannii Virulence
Source: PLoS One. 2015 Sep 24;10(9):e0138265. doi: 10.1371/journal.pone.0138265 (PMC4581634; doi:10.1371/journal.pone.0138265)
Supplement: S1 Table — (PDF) [file pone.0138265.s006.pdf]

S1 Table. Strains and plasmids included in this study.

Designation	Relevant characteristics	Reference or source
**Strains**		
A baumannii		
ATCC17978	Reference strain	ATCC
17978 Δ*tssM*	ATCC17978 isogenic derivative *tssM* unmarked deletion mutant	This study
17978 mini-CTX1	ATCC17978 carrying a chromosomal insertion of mini-CTX1 confering tetracycline resistance.	This study
DSM30011		ATCC
DSM30011 Δ*tssM*	DSM30011 isogenic derivative *tssM* deletion mutant, marked with an apramycin resistance cassette	This study
DSM30011 Δ*tssM* p*tssM*	DSM30011 Δ*tssM* complemented strain	This study
DSM30011 mini-Tn7	DSM30011 carrying a chromosomal insertion of mini Tn7 conferring gentamycin resistance.	This study
Ab242	Clinical isolate	[1]
Ab244	Clinical isolate	[1]
Ab825	Clinical isolate	[1]
E. coli		
DH5α	intermediate host for cloning and nalidixic acid resistant prey in bacterial competition assays. In some cases this strain contained a plasmid carrying apramycin cassette	[2]
P. aeruginosa		
PAK	prey in the bacterial competition assays.	[3]
PAK *retS*	*retS* mutant used as prey in the bacterial competition assays	[3]
PA14	prey in the bacterial competition assays and positive control in cell death experiments	
PA7	prey in the bacterial competition assays	[4]
K. pneumoniae		
52145	prey in the bacterial competition assays	[5]
**Plasmids**		
pGEM®-T Easy		Promega
pGEM-2.1	pGEM®-T Easy derivative carrying 2 kb upstream and downstream regions of DSM30011 *tssM* gene	This study
pGEM-2.2	pGEMT-2.1 derivative with DSM30011 *tssM* gene flanking regions interrupted by an apramycin resistant cassette	This study
pEX100T/Kan	pEX100T derivative carrying a kanamycin resistant cassette	[6]
pEX-TssM17	pEX100T/Kan derivative carrying 0.5 kb upstream and downstream regions of 17978 *tssM* gene	This study
pX5K	derivative of the RSF1010-derived plasmid pMM2017-Kan14	[7]
pTssM	pX5K derivative carrying DSM30011 *tssM* gene under the control of a constitutive promoter	This study

pMini Tn7	Used to insert gentamycin resistance into the chromosome for bacterial competition assays	[8]
pMini CTX1	Used to insert tetracyclin resistance into the chromosome for bacterial competition assays	[9]

1. Mussi MA, Limansky AS, Viale AM (2005) Acquisition of resistance to carbapenems in multidrug-resistant clinical strains of Acinetobacter baumannii: natural insertional inactivation of a gene encoding a member of a novel family of beta-barrel outer membrane proteins. Antimicrob Agents Chemother 49: 1432–1440. doi:10.1128/AAC.49.4.1432-1440.2005.

2. Hanahan D (1983) Studies on transformation of Escherichia coli with plasmids. J Mol Biol 166: 557–580.

3. Moscoso JA, Mikkelsen H, Heeb S, Williams P, Filloux A (2011) The Pseudomonas aeruginosa sensor RetS switches type III and type VI secretion via c-di-GMP signalling. Environ Microbiol 13: 3128–3138. doi:10.1111/j.1462-2920.2011.02595.x.

4. Cadoret F, Ball G, Douzi B, Voulhoux R (2014) Txc, a new type II secretion system of Pseudomonas aeruginosa strain PA7, is regulated by the TtsS/TtsR two-component system and directs specific secretion of the CbpE chitin-binding protein. J Bacteriol 196: 2376–2386. doi:10.1128/JB.01563-14.

5. March C, Moranta D, Regueiro V, Llobet E, Tomás A, et al. (2011) Klebsiella pneumoniae outer membrane protein A is required to prevent the activation of airway epithelial cells. Journal of Biological Chemistry 286: 9956–9967. doi:10.1074/jbc.M110.181008.

6. Schweizer HP, Hoang TT (1995) An improved system for gene replacement and xylE fusion analysis in Pseudomonas aeruginosa. Gene 158: 15–22.

7. Segal G, Shuman HA (1998) Intracellular multiplication and human macrophage killing by Legionella pneumophila are inhibited by conjugal components of IncQ plasmid RSF1010. Mol Microbiol 30: 197–208.

8. Choi K-H, Schweizer HP (2006) mini-Tn7 insertion in bacteria with single attTn7 sites: example Pseudomonas aeruginosa. Nature Protocols 1: 153–161. doi:10.1038/nprot.2006.24.

9. Hoang TT, Karkhoff-Schweizer RR, Kutchma AJ, Schweizer HP (1998) A broad-host-range Flp-FRT recombination system for site-specific excision of chromosomally-located DNA sequences: application for isolation of unmarked Pseudomonas aeruginosa mutants. Gene 212: 77–86.
