# Supplementary material for: Differential Role of the T6SS in Acinetobacter baumannii Virulence
Source: PLoS One. 2015 Sep 24;10(9):e0138265. doi: 10.1371/journal.pone.0138265 (PMC4581634; doi:10.1371/journal.pone.0138265)
Supplement: S2 Table — (PDF) [file pone.0138265.s007.pdf]

S2 Table. Primers used in this study

Name	Sequence (5' to 3')	Restriction site
17UpF	CTGTTATCCCTACCCGGGGCATTTTCCTGAACATATCG	SmaI
17UpR	GCTCTTTACGCTGTGCACTATCCTCATATAGTGCTGCGTTCC	-
17DoF	GATAGTGCACAGCGTAAAGAGC	-
17DoR	TAACAGGGTAATCCCGGGCATGTTCTCTTGTGGATTTGC	SmaI
17ChUp	GGATGGTCAAAGATTATATTCC	-
17ChDo	CACATTCACGTTCAGATAAAACC	-
DSMUpF	CGATACCCGGGGTTGAGTTGTATTTTTAAGC	SmaI
DSMUpR	CTGGATATCGAGTTTTGGCAGGATCAGCTGCCCTATTCTCGTTC TAAGTTCAAAG	EcoRV
DSMDoF	GGCAGCTGCTCCTGCCAAAACTGGATATCCAGTTTCTGC	EcoRV
DSMDoR	CGATACCCGGGGATAATGAATCATTTACTCTTGC	SmaI
ApraF	GCATCGATATCATCAAGGCCCGATCCTTGGAGCCCTTG	EcoRV
ApraR	GCATCGATATCTCATGAGCTCAGCCAATCGACTGG	EcoRV
CTssMF2	ATATGGATCCATGCATACAATTTTAGGCTACTTGTGGCAG	BamHI
CTssMR2	ATATGGATCCTCATGGCTTAACTCCCGCAGCAG	BamHI
T6ChUp	ATGCATGGGTCGATCCAAATAGCG	-
T6ChDo	GTAACAACCCAATTAATACTTCGG	-
HcpF	GAAAGATATATACGTTGAGTTTCG	-
HcpR	CTGCGTAAGAAGCTGTATTATTAG	-
TssMF	GCAAAGACGTCTACAACAGTTAGAC	-
TssMR	TTGCTGATCATCACGTTTACGAAC	-
16SF	GTGAGTAATGCTTAGGAATCTG	-
16SR	CATACTCTAGCTCACCAGTATC	-
