# Supplementary material for: Differential Role of the T6SS in Acinetobacter baumannii Virulence
Source: PLoS One. 2015 Sep 24;10(9):e0138265. doi: 10.1371/journal.pone.0138265 (PMC4581634; doi:10.1371/journal.pone.0138265)
Supplement: S3 Table — (PDF) [file pone.0138265.s008.pdf]

S3 Table. T6SS locus genes in *A. baumannii* strains under study.

	DSM30011		ATCC17978			Ab242/244/825	
**Protein Description**	**Locus**	**Length (AA)**	**Locus**	**Accession**	**Length (AA)**	**Locus**	**Length (AA)**
Conserved hypothetical (Signal peptide)	11700*	229	A1S_1292	YP_001084322	230	Ab244_01	230
TssB	11695	167	A1S_1293	YP_001084323	49	*tssB*	167
			A1S_1294	YP_001084324	79		
TssC	11690	493	A1S_1295	YP_001084325	443	*tssC*	493
TssD/Hcp	11685	167	A1S_1296	YP_001084326	167	*hcp*	167
TssE	11680	158	A1S_1297	YP_001084327	158	*tssE*	158
TssF	11675	603	A1S_1298	YP_001084328	185	*tssF*	603
			A1S_1299	YP_001084329	231		
TssG	11670	332	A1S_1300	YP_001084330	298	*tssG*	332
Putative membrane protein	11665*	470	A1S_1301	YP_001084331	421	Ab244_02	470
TssM	11660	1274	A1S_1302	YP_001084332	1041	*tssM*	1274
			A1S_1303	YP_001084333	188		
TagF	11655	319	A1S_1304	YP_001084334	275	*tagF*	319
TagN	11650	255	A1S_1305	YP_001084335	209	*tagN*	255
PAAR domain containing-protein	11645	87	A1S_1306	YP_001084336	87	Ab244_03	87
TssH/ClpV domain	11640	892	A1S_1307	YP_001084337	896	*tssH*	896
TssA	11635	364	A1S_1308	YP_001084338	273	*tssA*	364
TssK	11630	454	A1S_1309	YP_001084339	388	*tssK*	454
TssL	11625	268	A1S_1310	YP_001084340	268	*tssL*	268
Hypothetical protein	11620*	200	A1S_1311	YP_001084341	85	Ab244_04	200
Putative D-Ala D-Ala Carboxypeptidase	11615*	317	A1S_1312	YP_001084342	127	Ab244_05	317

* genes detected only in species of the genus *Acinetobacter*
