# Supplementary material for: Differential Role of the T6SS in Acinetobacter baumannii Virulence
Source: PLoS One. 2015 Sep 24;10(9):e0138265. doi: 10.1371/journal.pone.0138265 (PMC4581634; doi:10.1371/journal.pone.0138265)
Supplement: S4 Table — (PDF) [file pone.0138265.s009.pdf]

S4 Table. Putative VrgG proteins encoded by the *A. baumannii* strains under study.

Strain	VgrG protein (aa length)	Locus or GenBank accession number
DSM30011	932	KT334325
	875	KT334328
	1106	KT334326
	921	KT334327
ATCC17978	933	A1S_3364
	426[a]	A1S_1288
	395[b]	A1S_1289
	914*	A1S_0082
	921	A1S_0550
Ab244	788	AKR15126
	1104	AKR15129
	463[a]	AKR15122
	601[b]	AKR15123
	1064	AKR15127

[a]Proteins encoding VgrG N-terminal domain only.
[b]Proteins encoding VgrG C-terminal domain only.
*Annotated as a pseudogene .
